# Deazaflavin metabolite produced by endosymbiotic bacteria controls fungal host reproduction

**DOI:** 10.1093/ismejo/wrae074

**Published:** 2024-05-01

**Authors:** Ingrid Richter, Mahmudul Hasan, Johannes W Kramer, Philipp Wein, Jana Krabbe, K Philip Wojtas, Timothy P Stinear, Sacha J Pidot, Florian Kloss, Christian Hertweck, Gerald Lackner

**Affiliations:** Department of Biomolecular Chemistry, Leibniz Institute for Natural Product Research and Infection Biology (Leibniz-HKI), 07745 Jena, Thuringia, Germany; Junior Research Group Synthetic Microbiology, Leibniz Institute for Natural Product Research and Infection Biology (Leibniz-HKI), 07745 Jena, Thuringia, Germany; Department of Biomolecular Chemistry, Leibniz Institute for Natural Product Research and Infection Biology (Leibniz-HKI), 07745 Jena, Thuringia, Germany; Department of Biomolecular Chemistry, Leibniz Institute for Natural Product Research and Infection Biology (Leibniz-HKI), 07745 Jena, Thuringia, Germany; Department of Biomolecular Chemistry, Leibniz Institute for Natural Product Research and Infection Biology (Leibniz-HKI), 07745 Jena, Thuringia, Germany; Transfer Group Anti-Infectives, Leibniz Institute for Natural Product Research and Infection Biology (Leibniz-HKI), 07745 Jena, Thuringia, Germany; Department of Microbiology and Immunology, Doherty Institute, University of Melbourne, 3010 Melbourne, Victoria, Australia; Department of Microbiology and Immunology, Doherty Institute, University of Melbourne, 3010 Melbourne, Victoria, Australia; Transfer Group Anti-Infectives, Leibniz Institute for Natural Product Research and Infection Biology (Leibniz-HKI), 07745 Jena, Thuringia, Germany; Department of Biomolecular Chemistry, Leibniz Institute for Natural Product Research and Infection Biology (Leibniz-HKI), 07745 Jena, Thuringia, Germany; Faculty of Biological Sciences, Friedrich Schiller University Jena, 07743 Jena, Thuringia, Germany; Cluster of Excellence Balance of the Microverse, Friedrich Schiller University Jena, 07743 Jena, Thuringia, Germany; Junior Research Group Synthetic Microbiology, Leibniz Institute for Natural Product Research and Infection Biology (Leibniz-HKI), 07745 Jena, Thuringia, Germany; Chair of Biochemistry of Microorganisms, Faculty of Life Sciences: Food, Nutrition and Health, University of Bayreuth, 95326 Kulmbach, Bavaria, Germany

**Keywords:** host control, Mycetohabitans, Rhizopus microsporus, sporulation, endosymbiosis

## Abstract

The endosymbiosis between the pathogenic fungus *Rhizopus microsporus* and the toxin-producing bacterium *Mycetohabitans rhizoxinica* represents a unique example of host control by an endosymbiont. Fungal sporulation strictly depends on the presence of endosymbionts as well as bacterially produced secondary metabolites. However, an influence of primary metabolites on host control remained unexplored. Recently, we discovered that *M. rhizoxinica* produces F_O_ and 3PG-F_420_, a derivative of the specialized redox cofactor F_420_. Whether F_O_/3PG-F_420_ plays a role in the symbiosis has yet to be investigated. Here, we report that F_O_, the precursor of 3PG-F_420_, is essential to the establishment of a stable symbiosis. Bioinformatic analysis revealed that the genetic inventory to produce cofactor 3PG-F_420_ is conserved in the genomes of eight endofungal *Mycetohabitans* strains. By developing a CRISPR/Cas-assisted base editing strategy for *M. rhizoxinica*, we generated mutant strains deficient in 3PG-F_420_ (*M. rhizoxinica* Δ*cofC*) and in both F_O_ and 3PG-F_420_ (*M. rhizoxinica* Δ*fbiC*). Co-culture experiments demonstrated that the sporulating phenotype of apo-symbiotic *R. microsporus* is maintained upon reinfection with wild-type *M. rhizoxinica* or *M. rhizoxinica* Δ*cofC*. In contrast, *R. microsporus* is unable to sporulate when co-cultivated with *M. rhizoxinica* Δ*fbiC*, even though the fungus was observed by super-resolution fluorescence microscopy to be successfully colonized. Genetic and chemical complementation of the F_O_ deficiency of *M. rhizoxinica* Δ*fbiC* led to restoration of fungal sporulation, signifying that F_O_ is indispensable for establishing a functional symbiosis. Even though F_O_ is known for its light-harvesting properties, our data illustrate an important role of F_O_ in inter-kingdom communication.

## Introduction

Bacterial–fungal interactions are critically important in agriculture and human health, and their relevance to ecology is now seen as the norm [1], with numerous studies focusing on identifying the individual microorganisms residing within bacterial–fungal communities (e.g. The Human Microbiome Project) [2]. Among these interactions, fungal–bacterial endosymbiosis, in which bacteria reside within the cytosol of fungal hyphae, represents the most intimate relationship. Contemporary studies have revealed that endosymbiotic bacteria, commonly found in members of the *Mucoromycota*, are not unassuming background actors, but rather have considerable influence on fungal pathogenicity, physiology, and ecology [3].

**Figure 1:**
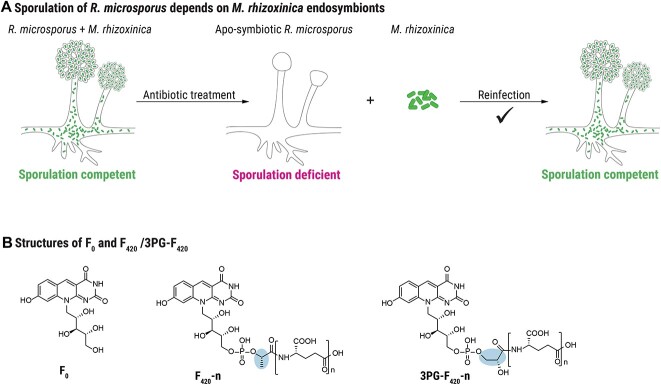
(A) Schematic representation of *M. rhizoxinica*-dependent sporulation of *R. microsporus*; mature sporangia are absent in fungi cured of endosymbionts by antibiotic treatment (apo-symbiotic *R. microsporus*); co-cultivation of axenic wild-type *M. rhizoxinica* with the apo-symbiotic fungus leads to recolonization, and the host’s ability to form sporangia is restored; (B) chemical structure of F_O_, F_420_, and 3PG-F_420_; *n* indicates the number of γ-linked glutamate residues.

The endosymbiosis between the *Mucoromycota* fungus *Rhizopus microsporus* and the bacterium *Mycetohabitans rhizoxinica* is a unique example of fungal pathogenicity being effectuated by an endosymbiont [4]. In housing *M. rhizoxinica*, *R. microsporus* gains the ability to kill rice seedlings through the secretion of the bacterial secondary metabolite rhizoxin [5], which also protects the fungus from soil-dwelling micropredators [6]. A fundamental process that is key for the persistence of the *Rhizopus*–*Mycetohabitans* symbiosis is that endosymbionts are translocated into the fungal spores during host reproduction [7]. If endosymbionts are eliminated through antibiotic treatment, *R. microsporus* is unable to reproduce vegetatively (Fig. 1A) [7]. Thus, the ability of *R. microsporus* to reproduce asexually via sporulation strictly depends on the presence of endobacteria. This endosymbiont-dependent control of fungal sporulation is known to be mediated by a multitude of bacterially produced symbiosis factors such as a specialized lipopeptide O-antigen [8], transcription activator-like effectors [9], and the secondary metabolite habitasporin [10].

Even though *M. rhizoxinica* is known for its high potential for secondary metabolite biosynthesis [10, 11], it also produces unusual molecules linked to primary metabolism such as F_O_ (7,8-didemethyl-8-hydroxy-5-deazariboflavin) and 3PG-F_420_, a derivative of the deazaflavin cofactor F_420_ (Fig. 1B) [12]. Although F_O_ is the precursor of 3PG-F_420_, both metabolites have entirely distinct physiological roles. F_O_ plays a key role as a light-harvesting chromophore in DNA photolyases across all three domains of life (*Bacteria*, *Archaea*, and *Eukarya*) [13–15]. F_420_ was first discovered as a cofactor of methanogenesis in anaerobic archaea [16]. Beyond archaea, F_420_ metabolism has only been extensively studied in the bacterial phylum *Actinobacteria* [17], where it mediates diverse catalytic reactions central to processes such as antibiotic biosynthesis in streptomycetes [18] and drug resistance in *Mycobacterium tuberculosis* [19]. More recently, genomic studies uncovered a broad distribution of genes encoding enzymes responsible for the biosynthesis of F_420_ in Gram-negative bacteria [20, 21]. Of these, three species were confirmed as F_420_ producers [21].

F_420_ production by Gram-negative bacteria that engage in a symbiotic lifestyle has been reported in two cases—namely, the sponge symbiont *Candidatus* Entotheonella factor [22] and *M. rhizoxinica* [12]. So far, the physiological role of the deazaflavin cofactor in these microorganisms remains unknown. The *R. microsporus*–*M. rhizoxinica* symbioses provide an ideal model system to investigate the function of F_420_ because *M. rhizoxinica* can be cultured axenically and a readily discernible phenotype exists that is indicative of a stable symbiosis, i.e. sporulation of the fungal host. Other than the discernment of whether metabolites from *M. rhizoxinica* contribute to the ability of *R. microsporus* to reproduce, the *Rhizopus*–*Mycetohabitans* model allows earlier aspects of symbiosis establishment to be probed such as bacterial colonization of hyphae and intracellular distribution and survival [23–26].

Here, we show that homologous genes encoding the pathway to produce 3PG-F_420_ are conserved in the genomes of several endofungal *Mycetohabitans* symbionts. Furthermore, we demonstrate that F_O_, the precursor of 3PG-F_420_, is a symbiosis factor that is instrumental in the maintenance of the phytopathogenic *R. microsporus–M. rhizoxinica* alliance.

## Materials and methods

### Strains and culturing conditions

Eight *R. microsporus* strains harboring *Mycetohabitans* spp. endobacteria were used in this study (Supplementary Table 1) [27]. Endobacteria from *R. microsporus* ATCC62417 were eliminated by continuous antibiotic treatment [28] resulting in apo-symbiotic *R. microsporus* (RMapo). The absence of endobacteria was confirmed by fluorescence microscopy and an absence of rhizoxin in extracts of the fungal mycelium [5]. Both *R. microsporus* strains (ATCC62417 and RMapo) were cultivated on Potato Dextrose Agar (PDA; Becton, Dickinson & Company, Sparks, MD) at 30°C. Bacterial endosymbionts were isolated from the mycelium of eight fungal strains as previously reported [29]. Pure cultures of *M. rhizoxinica* were grown at 30°C in MGY M9 minimal medium (10 g/l glycerol, 1.25 g/l yeast extract, M9 salts: 7 g/l K_2_HPO_4_, 2 g/l KH_2_PO_4_, 600 mg/l C_6_H_7_NaO_7_, 1 g/l (NH_4_)_2_SO_4_, and 100 mg/l Mg_2_SO_4_) or Standard I Nutrient Agar (Merck, Darmstadt, Germany) supplemented with 1% glycerol.

### Gene expression studies

RNA was isolated from *M. rhizoxinica* HKI-454 using the Quick-RNA Fungal/Bacterial Miniprep Kit (Zymo Research, Irvine, CA) following the manufacturers’ recommendations. Because there is no standard method for RNA extraction from endofungal bacteria, we developed our own protocol for reliable RNA extraction from symbiotic *M. rhizoxinica* (see Supplementary File “Materials and Methods” for details).

**Figure 2:**
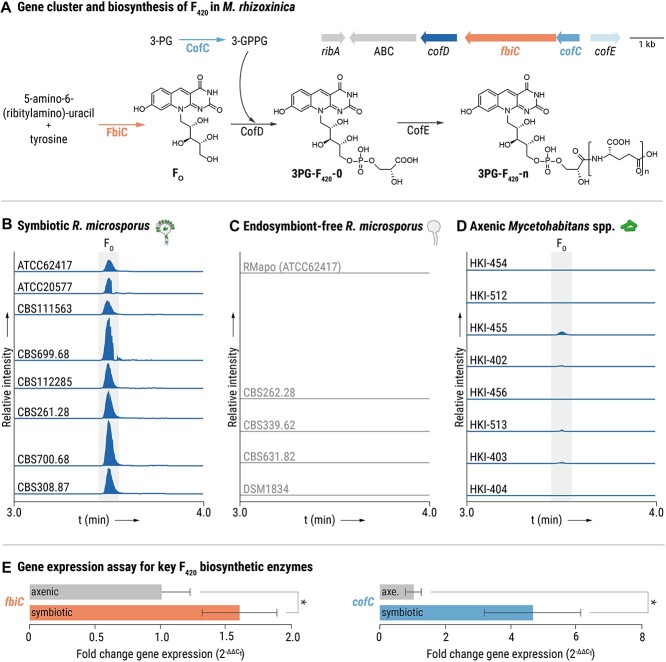
Products of conserved F_420_ biosynthetic gene clusters are produced under symbiotic conditions; (A) representative F_420_ biosynthetic gene cluster detected in endofungal *Mycetohabitans* strains (abbreviations: *ribA*, GTP cyclohydrolase II; ABC, ABC transporter involved in heavy metal resistance; *cofD*, 3-phospho-d-glycerate transferase; *fbiC*, Fo synthase; *cofC*, 3-phospho-d-glycerate guanylyltransferase; *cofE,* F_420_-0:L-glutamate ligase; Supplementary Fig. 1) and biosynthesis of 3PG-F_420_-n in *M. rhizoxinica* [15]; (B) extracted ion chromatograms (EICs, 5 ppm mass window) of F_O_ ([M+H]^+^*m/z* 364.11393) produced by eight *R. microsporus* strains containing *Mycetohabitans* spp. endosymbionts; (C) EICs of F_O_ produced by *R. microsporus* (ATCC62417) cured of its endosymbionts (RMapo) and by *R. microsporus* strains that are naturally endosymbiont-free; (D) EICs of F_O_ produced by axenic *Mycetohabitans* spp. strains that were isolated from their corresponding *R. microsporus* host; (E) gene expression assay for two F_420_ biosynthetic enzymes; the expression level of genes *fbiC* and *cofC* in *M. rhizoxinica* was measured in pure culture (axenic) and in symbiosis with *R. microsporus* (symbiotic) using qPCR; the gene *rpoB* was used to calculate expression levels using the 2^–ΔΔCt^ method; bars represent means of three independent biological replicates (*n* = 3) and error bars indicate standard deviation; unpaired *t*-test with Welch’s correction (^*^P<05, Supplementary Tables 5 and 6).

Quantitative PCR (qPCR) was used to study expression levels of two genes (*fbiC* and *cofC*) in axenic *M. rhizoxinica* HKI-454 as well as in *M. rhizoxinica* HKI-454 living in symbiosis with *R. microsporus* (ATCC62417). The gene *fbiC* encodes F_O_ synthase, which catalyzes the key step in F_420_ biosynthesis leading to the formation of the metabolically active precursor F_O_ (Fig. 2A) [30]. A second important step, the side-chain biosynthesis of 3PG-F_420_-0 is catalyzed by CofC. The *M. rhizoxinica rpoB* gene was used as an internal control for calculation of expression levels and normalization. All qPCR primer pairs used are listed in Supplementary Table 2A. First, primer efficiencies were calculated from standard curves generated with serial dilutions (ranging from 1 to 10^−3^) of axenic *M. rhizoxinica* cDNA. All primer pairs have an efficiency of 93% to 96% and were used in subsequent gene expression experiments (Supplementary Table 3) using My*Taq* HS Mix (Bioline, London, UK) and EvaGreen® Fluorescent DNA Stain (Jena Bioscience, Jena, Germany). Each sample was run in three technical replicates on a QuantStudio 5 Real-Time PCR machine (Applied Biosystems, Waltham, MA). A control reaction, in which sterile water replaced the cDNA, was carried out in parallel. Amplifications of each template were performed in three biological replicates (*n* = 3). The cycle threshold (C_t_) values were calculated using the Design and Analysis Software v1.5.2 (Applied Biosystems). C_t_ values were used for quantification of expression levels via the 2^−ΔΔCt^ method [31] in MS Excel. Statistical analysis was performed in GraphPad Prism 9.5.1 (GraphPad Software, La Jolla, CA, www.graphpad.com). An unpaired *t*-test with Welch’s correction was used to study the gene expression level of *fbiC* and *cofC* in *M. rhizoxinica* wild type grown in axenic culture in relation to *M. rhizoxinica* living in symbiosis with *R. microsporus*.

### Extraction of F_O_/3PG-F_420_-n from fungal mycelium and axenic *Mycetohabitans* spp.

Small pieces of fungal mycelium were inoculated in 100 ml nutrient broth medium (Merck Millipore, Darmstadt, Germany) in 500 ml baffled Erlenmeyer flasks and incubated at 30°C and 110 rpm. After 7 days of incubation, fungal mycelium was strained through a 40 μm cell strainer (Corning Inc.) and resuspended in 10 ml ice-cold HPLC-grade methanol (VWR Chemicals, Darmstadt, Germany). Axenic bacterial overnight cultures of *Mycetohabitans* spp. were inoculated in 50 ml MGY M9 minimal medium in 300 ml baffled Erlenmeyer flasks and cultured at 30°C and 110 rpm. After 7 days of incubation, bacterial cultures were snap-frozen in liquid nitrogen in 500 ml round-bottom flasks and freeze-dried overnight (Christ Alpha loc-1 m, Martin Christ Gefriertrocknungsanlagen GmbH, Osterode am Harz, Germany). Dry *Mycetohabitans* spp. cultures were then resuspended in 10 ml ice-cold HPLC-grade methanol (VWR Chemicals).

All resuspended samples were sonicated for 20 min (Sonorex RK100 Ultrasonic bath, Bandelin, Berlin, Germany) and then shaken (250 rpm) for 1 h followed by centrifugation (10 000 × *g*) for 15 min (Centrifuge 5810R, Eppendorf, Hamburg, Germany). The supernatant was filtered through a paper filter into a 500 ml round-bottom flask and the solvent was evaporated on a vacuum rotary evaporator (Laborota 4003, Heidolph, Schwabach, Germany). Dry extracts were redissolved in 2 ml LC–MS-grade water (Carl Roth, Karlsruhe, Germany). The aqueous extracts were loaded on to a 100 mg C18 Chromabond cartridge (Macherey-Nagel, Düren, Germany), which was washed with methanol and conditioned with LC–MS-grade water beforehand. The cartridge was then washed and desalted with 1 ml LC–MS-grade water, followed by elution of the sample in three fractions with methanol (0.5 ml, 1.0 ml, and 0.5 ml). F_O_ was almost always concentrated in the second fraction. The samples were centrifuged at 10000 × *g* for 20 min and the supernatant was stored at −20°C until further LC–MS analysis (see Supplementary File “Materials and Methods” for details).

### Generation of *M. rhizoxinica* Δ*fbiC* and *M. rhizoxinica* Δ*cofC* using CRISPR/Cas cytidine base editing and subsequent genetic complementation

To investigate a possible role of F_O_ and 3PG-F_420_-n in the symbiosis, two genes (*fbiC* and *cofC*) were deleted using cytidine base editing. To establish a working CRISPR/Cas cytidine base editing method for *M. rhizoxinica* HKI-454, we used our knowledge gained from the CRISPR/Cas modification of the Betaproteobacterium *Burkholderia gladioli* [32], where we utilized a temperature-sensitive, low-copy plasmid (pTsK-CasRed-Bt, Supplementary Table 4) containing among others a codon-optimized cas9^*^ gene. See Supplementary File “Materials and Methods” for details on the generation of the base editing plasmids pTsK-AnCU-fbiC (targeting *fbiC*) and pTsK-AnCU-cofC (targeting *cofC*) and subsequent transfer of the plasmids into *M. rhizoxinica* by electroporation.

Both *M. rhizoxinica* Δ*fbiC* and *M. rhizoxinica* Δ*cofC* were checked for deleterious mutations using whole genome sequencing (see Supplementary File “Materials and Methods” for details). Both mutants were subsequently genetically complemented. Briefly, *M. rhizoxinica* Δ*fbiC* and *M. rhizoxinica* Δ*cofC* were transformed with the plasmids pRANGER-*fbiC* and pRANGER-*cofC*, respectively. These expression vectors carry the native promoter controlling expression of each gene, yielding the complemented strains *M. rhizoxinica* Δ*fbiC* pRANGER-*fbiC* and *M. rhizoxinica* Δ*cofC* pRANGER-*cofC* (see Supplementary File “Materials and Methods” for details).

### Co-culture / sporulation bioassay

Liquid sporulation bioassays, containing apo-symbiotic *R. microsporus* and 100 μl of overnight cultures of *M. rhizoxinica* wild type (HKI-454), *M. rhizoxinica* Δ*fbiC*, *M. rhizoxinica* Δ*cofC*, *M. rhizoxinica* Δ*fbiC* pRANGER-*fbiC*, or *M. rhizoxinica* Δ*cofC* pRANGER-*cofC* were performed as previously described [9]. Experiments were performed at least four times independently (*n* ≥ 4 biological replicates) with six technical replicates on each plate. GraphPad Prism 9.5.1 (GraphPad Software) was used for statistical analysis and graphing. Data from spore counts were compared between *M. rhizoxinica* strains using one-way analysis of variance (ANOVA) and Tukey Honestly Significant Difference (HSD) test function in GraphPad. *P* values (*P*) *<*.05 were considered statistically significant. The Brown–Forsythe test was used to test for equal variance and a *P* < .05 was considered significant.

To test whether F_O_ alone could trigger fungal sporulation or whether the presence of bacteria is still necessary, we chemically synthesized F_O_ (see Supplementary File “Materials and Methods” for details) and added varying concentrations of synthetic F_O_ (312 ng/ml, 625 ng/ml, and 937 ng/ml) to co-cultures of *R. microsporus* and *M. rhizoxinica* Δ*fbiC*. Apo-symbiotic *R. microsporus* was also grown in the absence of endobacteria in 48-well plates in either liquid or solid potato dextrose medium that was supplemented with a final concentration of 73 μg/ml synthetic F_O_. Spores were harvested and quantified as described previously [9], and the formation of sporangia was visualized using a Zeiss Axio Zoom.V16 Stereomicroscope (Carl Zeiss Microscopy, Oberkochen, Germany).

### Imaging of endohyphal *M. rhizoxinica* strains

To visualize the localization of F_420_-producing and F_420_-deficient *M. rhizoxinica* strains within the fungal hyphae, strains were transformed with a plasmid encoding green fluorescent protein (GFP) (see Supplementary File “Materials and Methods” for details) and then used in the liquid sporulation assays as described previously [9]. After 5 days of incubation, fungal mycelium was transferred to PDA Petri dishes. Sterile, high-performance cover glasses (D = 0.17 mm +/− 0.005 mm, refractive index = 1.5255 +/− 0.0015, Carl Zeiss Microscopy) were placed on the agar plate in close proximity to the mycelium. Plates were incubated for 1–2 days at 30°C to allow fungal mycelium to grow on top of the cover glass. Once fungal mycelium had grown on top of the cover glass, the cover glasses were carefully removed from agar plates, and the mycelium was fixed for 5 min in fixing solution (3.7% formaldehyde, 25 mM KH_2_HPO_4_). Samples were washed at least three times with 25 mM KH_2_HPO_4_ before mounting the cover slide in embedding agent (ProLong Gold Antifade Mountant, Invitrogen) on a microscope slide (superfrost, ground 90°, Thermo Scientific). Fluorescence microscopy was carried out using a fluorescence wide-field microscope (LaserWF mode in the ELYRA 7, Carl Zeiss Microscopy), and bacterial cells were visualized using a laser excitation at 488 nm and an emission bandpass from 495 nm to 590 nm. For superresolution images, the structured illumination mode was used with a grating of 27.5 μm period. Fluorescent wide-field images were used to quantify bacterial cells inside of fungal hyphae (see Supplementary File “Materials and Methods” for details).

## Results

### Products of conserved F_420_ biosynthetic gene clusters are produced under symbiotic conditions

The discovery that two *Mycetohabitans* strains encode a complete F_420_ biosynthetic gene cluster [12, 33], despite their highly reduced genomes [10, 34], raised the question as to whether the cofactor might contribute to the integrity of the bacterial–fungal symbiosis. Therefore, we searched the genomes of six additional *Mycetohabitans* strains that are endosymbionts of globally distributed *R. microsporus* strains for F_420_ biosynthesis loci (Supplementary Table 1) [27]. We found a complete, highly conserved F_420_ biosynthesis locus, containing the requisite genes for F_420_ biosynthesis, in all *Mycetohabitans*-symbionts of *R. microsporus* (Fig. 2A and Supplementary Fig. 1). The gene *fbiC* encodes F_O_ synthase, which catalyzes the key step in F_420_ biosynthesis leading to the formation of the metabolically active precursor F_O_ (Fig. 2A) [30]. A second important step, the side-chain biosynthesis of 3PG-F_420_-0 is catalyzed by CofC and CofD [12, 33]. Finally, a γ-glutamyl ligase (CofE) catalyzes the successive addition of l-glutamate residues (n) to 3PG-F_420_-0 yielding 3PG-F_420_-n [35].

To investigate under which conditions 3PG-F_420_ and its precursor F_O_ are produced, we extracted and analyzed the following cultures by liquid chromatography coupled with tandem mass spectrometry (LC–MS/MS): (i) eight *R. microsporus* strains containing their corresponding *Mycetohabitans* spp. endosymbionts, (ii) one *R. microsporus* strain (ATCC62417) cured of its endosymbionts (RMapo), (iii) *R. microsporus* strains that are naturally endosymbiont-free, and (iv) axenic *Mycetohabitans* spp. strains that were isolated from their corresponding *R. microsporus* host. The metabolic profiling showed that both F_O_ and 3PG-F_420_-n species are predominantly produced in *Mycetohabitans* strains when living as endosymbionts of *R. microsporus* (Fig. 2B and Supplementary Fig. 2A). In contrast, apo-symbiotic and naturally endosymbiont-free *R. microsporus* strains do not produce any F_O_ or 3PG-F_420_-n species (Fig. 2C and Supplementary Fig. 2B). We noted that endosymbiotic bacteria grown outside of their host sporadically produce F_O_ and 3PG-F_420_-n under the conditions tested (Fig. 2D and Supplementary Fig. 2C). 3PG-F_420_-2 and 3PG-F_420_-3 are the most abundant species in *Mycetohabitans* strains, although up to four glutamate residues were detected (3PG-F_420_-4) (Supplementary Fig. 2C).

In light of the finding that F_O_ and 3PG-F_420_-n are predominantly produced under symbiotic conditions, we probed whether F_O_ and 3PG-F_420_-n production is constitutive or dependent on the presence of the fungal host. Gene expression of *fbiC* and *cofC* was monitored in axenic *M. rhizoxinica* HKI-454 and in *M. rhizoxinica* HKI-454 living as endosymbiont in *R. microsporus* ATCC62417. These genes were chosen because they both encode enzymes that catalyze key steps in F_420_ biosynthesis (Fig. 2A). A gene encoding the *β*-subunit of the bacterial RNA polymerase (*rpoB*) was used as an internal control for RNA integrity and cDNA synthesis efficiency. Although it is inherently difficult to isolate high-quality RNA from endofungal bacteria, we developed a reliable method for RNA extraction from symbiotic *M. rhizoxinica* (Supplementary Fig. 3). Using qPCR, we show that *fbiC* is 1.6-fold upregulated (unpaired *t*-test: *t* = 2.87, df = 4.0, *P =* .0455, Supplementary Table 5) and *cofC* is 4.6-fold upregulated (unpaired *t*-test: *t* = 4.221, df = 4.0, *P =* .0135, Supplementary Table 6) in symbiotic *M. rhizoxinica* compared to axenic bacterial cultures (Fig. 2E).

Based on the prevalence of F_420_-biosynthesis genes in the genomes of endofungal *Mycetohabitans* strains and the increased production of F_O_ and 3PG-F_420_-n under symbiotic conditions, backed up by elevated transcription levels of F_420_ biosynthetic genes, we deemed it important to further probe the role of these metabolites in the symbiosis.

### Generation of F_420_-deficient *M. rhizoxinica* using CRISPR/Cas-assisted base editing

To facilitate subsequent investigations into the role of F_O_/3PG-F_420_ in the *Rhizopus–Mycetohabitans* symbiosis, we performed targeted gene inactivation of *fbiC* and *cofC*. As both genes belong to one operon and are under the control of the same promotor (Fig. 2B), we intended to produce markerless gene inactivations to avoid possible polar effects. Specifically, our goal was to introduce a stop codon into the coding sequences of *fbiC* and *cofC*, creating two individual *M. rhizoxinica* mutant strains. We deemed cytidine base editing a promising method to introduce stop codons [36].

**Figure 3:**
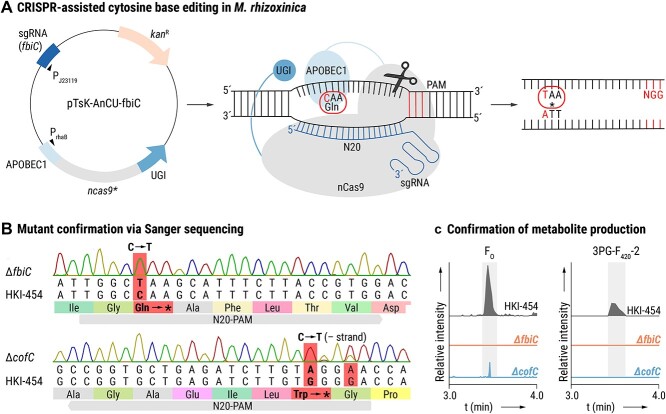
Generation of *M. rhizoxinica* Δ*fbiC* and *M. rhizoxinica* Δ*cofC* using CRISPR/Cas-assisted base editing; (A) schematic overview of the temperature-sensitive plasmid pTsK-AnCU-fbiC encoding a fusion protein consisting of a cytidine deaminase (APOBEC1), a Cas9-nickase (nCas9), and an UGI; the plasmid also carries the *fbiC*-specific sgRNA (left panel); APOBEC1 deaminates a cytosine (C) to uracil (U); the resulting mismatch leads to the replacement of guanosine (G) with adenosine (A) on the non-edited strand (middle panel); the artificially generated U-A base pair is converted to a T-A base pair during DNA replication; this leads to the conversion of glutamine to a stop codon (right panel); the plasmid pTsK-AnCU-cofC, carrying the *cofC*-specific sgRNA, was constructed in an analogous manner; Abbreviations: APOBEC1, rat cytidine deaminase; ncas9^*^, codon-optimized gene for Cas9 nickase from *S. pyogenes*; ; *kan*^R^, kanamycin resistance cassette; P_rhaB_, rhamnose-inducible promoter; P_J23119_, constitutive promoter; PAM, protospacer adjacent motif; N20, the 20-bp sequence at the 5′ end upstream of the PAM; Gln, glutamine; ^*^, stop codon; (B) verification of base editing in *M. rhizoxinica* Δ*fbiC* (exchange of C to T) and in *M. rhizoxinica* Δ*cofC* (exchange of C to T on the minus strand, resulting in an exchange of G to A on the complementary strand) via Sanger sequencing; (C) confirmation of metabolite production via LC-MS/MS analysis; EICs (5 ppm mass window) confirming the absence of both F_O_ ([M+H]^+^*m/z* 364.11393) and 3PG-F_420_-2 ([M+H]^+^*m/z* 790.18149) production by *M. rhizoxinica* Δ*fbiC*, the presence of F_O_ and absence of 3PG-F_420_-2 production by *M. rhizoxinica* Δ*cofC*, and the production of both metabolites by *M. rhizoxinica* wild type (HKI-454).

To generate base editing plasmids, a temperature-sensitive, low-copy plasmid containing a rhamnose-inducible and codon-optimized cas9^*^ gene was used [32]. We added a gene that encodes a fusion protein consisting of APOBEC1 (a cytidine deaminase from rats), nCas9 (Cas9-nickase with a D10A mutation from *Streptococcus pyogenes*), and a uracil DNA glycosylase inhibitor (UGI) from a *Bacillus* phage (Fig. 3A).

The base editing plasmids pTsK-AnCU-fbiC and pTsK-AnCU-cofC (Supplementary Table 4) differed only in the synthetic guide RNA (sgRNA) sequences in which the N20 sequences determine the individual target site. Both plasmids were used for precisely targeting *fbiC* and *cofC* in the *M. rhizoxinica* genome to generate the mutants *M. rhizoxinica* Δ*fbiC*::stop (*M. rhizoxinica* Δ*fbiC*) and *M. rhizoxinica* Δ*cofC*::stop (*M. rhizoxinica* Δ*cofC*), respectively. Successful base editing in potential mutants was verified via Sanger sequencing (Fig. 3B). Whole genome sequencing of *M. rhizoxinica* Δ*fbiC* and *M. rhizoxinica* Δ*cofC* revealed no deleterious mutations in either strain, thus confirming the specificity of the chosen sgRNA. CRISPR/Cas-mediated base editing was previously developed for *Gammaproteobacteria* (*Pseudomonas putida*) [37] and *Alphaproteobacteria* (*Brucella melitensis*) [38]. Here, we established this genome editing strategy for *Betaproteobacteria* (*M. rhizoxinica*).

As expected, metabolic profiling of the mutant strains using LC–MS/MS confirmed that F_O_ and 3PG-F_420_ biosynthesis is abolished in *M. rhizoxinica* Δ*fbiC*, whereas *M. rhizoxinica* Δ*cofC* is able to produce F_O_ but no 3PG-F_420_-2 (Fig. 3C). In comparison, *M. rhizoxinica* wild type produces F_O_ and 3PG-F_420_-2 in axenic culture under the same conditions. Axenically grown *M. rhizoxinica* Δ*fbiC* displays growth behavior similar to the wild type when cultivated on a selection of carbon sources in liquid media (Supplementary Fig. 4). A broader screen for altered utilization of carbon sources using phenotypic microarrays did not reveal any metabolic deficit of F_O_-deficient *M. rhizoxinica* (Supplementary Table 7).

### F_O_-deficient *M. rhizoxinica* abolish the sporulation ability of *R. microsporus*

**Figure 4:**
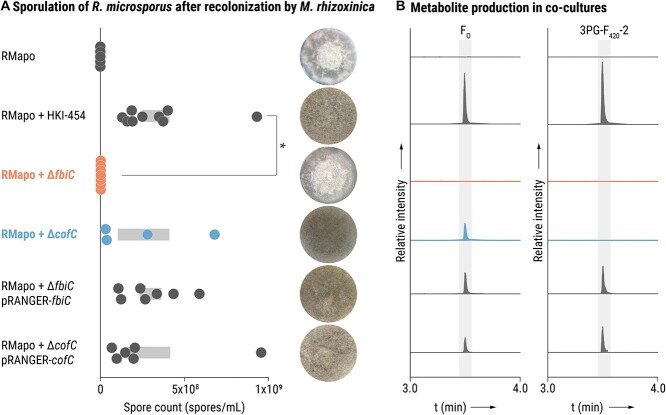
F_O_-deficient *M. rhizoxinica* do not trigger sporulation of *R. microsporus*; (A) spore count and photographs of apo-symbiotic *R. microsporus* incubated with solvent control (RMapo) or recolonized by *M. rhizoxinica* wild type (RMapo + HKI-454), or *M. rhizoxinica* F_420_ mutant strains (RMapo + *M. rhizoxinica* Δ*fbiC* and RMapo + *M. rhizoxinica* Δ*cofC*), or complemented *M. rhizoxinica* strains (RMapo + *M. rhizoxinica* Δ*fbiC* pRANGER-*fbiC* and RMapo + *M. rhizoxinica* Δ*cofC* pRANGER-*cofC*) after 1 week of co-cultivation; dots represent biological replicates (*n* ≥ 4) and grey bars mark ± one standard error of the mean (SEM); one-way ANOVA with Tukey’s multiple comparison test (^*^P<.05, Supplementary Table 8) was applied; (B) production of F_O_/3PG-F_420_-2 was confirmed by LC–MS/MS; EICs (5 ppm mass tolerance) of F_O_ ([M+H]^+^*m/z* 364.11393) and 3PG-F_420_-2 ([M+H]^+^*m/z* 790.18149) produced in either apo-symbiotic *R. microsporus* (RMapo) or in co-cultures of apo-symbiotic *R. microsporus* with *M. rhizoxinica* strains; strain order as given in Panel A.

The ability of *R. microsporus* to reproduce asexually via sporulation strictly depends on the presence of endobacteria as well as secondary metabolites produced by *M. rhizoxinica* [7, 10, 26]. Thus, considering that F_O_ and 3PG-F_420_ are predominantly produced under symbiotic conditions, we aimed to explore the potential involvement of 3PG-F_420_ and/or its precursor F_O_ in fungal sporulation. The sporulation ability of *R. microsporus* containing either *M. rhizoxinica* Δ*fbiC* or *M. rhizoxinica* Δ*cofC* was investigated in a sporulation bioassay [24]. Briefly, apo-symbiotic *R. microsporus* mycelium is co-cultured with axenic *M. rhizoxinica* wild type or mutant strains in 48-well plates. After 5–7 days of incubation, the formation of spores can be observed indicating the successful establishment of the symbiosis [7]. Apo-symbiotic *R. microsporus* does not sporulate (Fig. 4A). Subsequently, the absence/presence of F_O_ and 3PG-F_420_ in co-cultures was verified via LC–MS/MS analysis.

*M. rhizoxinica* Δ*cofC* is able to produce F_O_, but not 3PG-F_420_, and readily triggers fungal sporulation to a similar degree as the wild type. (Fig. 4A and B and Supplementary Table 8). In contrast, in the case of co-cultivation with *M. rhizoxinica* Δ*fbiC*, spores are absent and the sporulation efficiency is significantly reduced (*P <* .05) when compared to the *M. rhizoxinica* wild type (Fig. 4A and Supplementary Table 8). As expected, F_O_ and 3PG-F_420_ are absent in *R. microsporus*–*M. rhizoxinica* Δ*fbiC* co-cultures (Fig. 4B). Thus, the inability to sporulate appears to be linked to a lack of F_O_ but not 3PG-F_420_. In addition, growth of the fungal mycelium containing *M. rhizoxinica* Δ*fbiC* is slower and fewer aerial hyphae are formed, which is comparable to apo-symbiotic fungal mycelium (Fig. 4A). The observation that F_O_-deficiency completely abolishes *R. microsporus* sporulation is unexpected, as a complete abrogation of fungal sporulation is rarely observed [24].

To confirm that the inability of *M. rhizoxinica* Δ*fbiC* to induce fungal sporulation is solely due to disruption of the *fbiC* gene, we performed an *in vivo trans*-complementation experiment. To this end, we transformed *M. rhizoxinica* Δ*fbiC* and *M. rhizoxinica* Δ*cofC* with the plasmids pRANGER-*fbiC* and pRANGER-*cofC*, respectively. These expression vectors carry the native promoter controlling expression of each gene, yielding the complemented strains *M. rhizoxinica* Δ*fbiC* pRANGER-*fbiC* and *M. rhizoxinica* Δ*cofC* pRANGER-*cofC* (Supplementary Fig. 5). Recolonization of apo-symbiotic *R. microsporus* by Δ*cofC* pRANGER-*cofC* leads to a level of fungal sporulation similar to that imparted by the wild type (Fig. 4A). In addition, co-incubation of *R. microsporus* with Δ*fbiC* pRANGER-*fbiC* readily triggers sporulation, thus restoring the wild-type phenotype (Fig. 4A). As expected, both complemented strains are able to produce F_O_ and 3PG-F_420_-2 in co-culture with *R. microsporus* (Fig. 4B). These results show that a lack of 3PG-F_420_ alone does not affect the ability of *R. microsporus* to sporulate. However, when 3PG-F_420_-deficiency is coupled with a lack of F_O_, fungal sporulation is abolished, signifying that F_O_ is an essential mediator in the sporulation process of endosymbiont-dependent *R. microsporus*.

### Chemical complementation of F_O_-deficient *M. rhizoxinica* restores sporulation in *R. microsporus*

**Figure 5:**
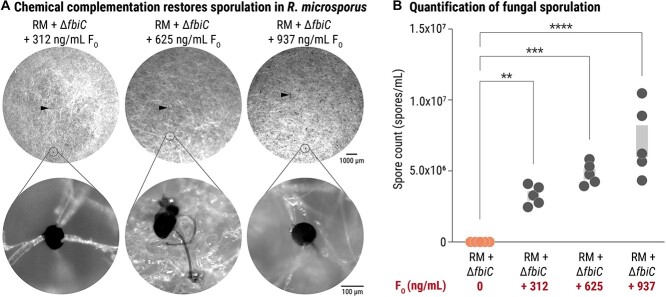
Chemical complementation with synthetic F_O_ restores the sporulation ability of *R. microsporus* containing F_O_-deficient *M. rhizoxinica*; (A) photographs and microscopic images of *R. microsporus–M. rhizoxinica* Δ*fbiC* co-cultures (RMapo + *M. rhizoxinica* Δ*fbiC*) supplemented with increasing concentrations of synthetic F_O_ (312 ng/ml, 625 ng/ml, and 937 ng/ml); formation of sporangia (representative examples marked by black arrows) was observed; close-up images show mature sporangia; (B) spore count of apo-symbiotic *R. microsporus* recolonized by *M. rhizoxinica* Δ*fbiC* (RMapo + *M. rhizoxinica* Δ*fbiC*) and supplemented with synthetic F_O_ after 1 week of co-cultivation; dots represent biological replicates (*n* = 5), grey bars mark ± one standard error of the mean (SEM); one-way ANOVA with Tukey’s multiple comparison test (^*^^*^P<.002, ^*^^*^^*^P<.0002, ^*^^*^^*^^*^P<.0001, Supplementary Table 9) was applied; data points of RMapo + Δ*fbiC* are the same as depicted in Fig. 4A.

The observation that F_O_-deficiency completely abolishes fungal sporulation raised the question as to whether F_O_ alone can trigger *R. microsporus* sporulation or whether the presence of bacteria is necessary for this process. To answer this question, chemical complementation with synthetic F_O_ was performed. Apo-symbiotic *R. microsporus* was co-incubated with *M. rhizoxinica* Δ*fbiC* in a sporulation assay as described above. In addition, synthetic F_O_ was added to individual wells containing apo-symbiotic *R. microsporus* and *M. rhizoxinica* Δ*fbiC* at varying concentrations (312 ng/ml, 625 ng/ml, or 937 ng/ml). When synthetic F_O_ is added to non-sporulating *R. microsporus*–*M. rhizoxinica* Δ*fbiC* co-cultures, spore formation is restored (Fig. 5A). Although the number of spores is lower than induced by the wild type, the sporulation in chemically complemented co-cultures is significantly increased (*P <* .002) when compared to *M. rhizoxinica* Δ*fbiC* (Fig. 5B and Supplementary Table 9). In fact, we observed a dose-dependent increase in the number of spores with increasing amounts of F_O_ supplementation (Fig. 5B and Supplementary Table 9).

As these results clearly confirmed the importance of F_O_ in fungal sporulation, we were curious as to whether F_O_ itself could induce sporulation in *R. microsporus* in the absence of endobacteria. Apo-symbiotic *R. microsporus* was grown in 48-well plates in either liquid or solid potato dextrose medium containing a final concentration of 73 μg/ml synthetic F_O_. Spore formation was not detected, even after 2 weeks of incubation (Supplementary Fig. 6). Together with the successful chemical complementation of *M. rhizoxinica* Δ*fbiC*, these results indicate that F_O_ alone is not sufficient to trigger sporulation of the host fungus and that other bacterial traits are involved in this process.

### F_O_-deficient *M. rhizoxinica* are able to recolonize *R. microsporus* hyphae efficiently

**Figure 6:**
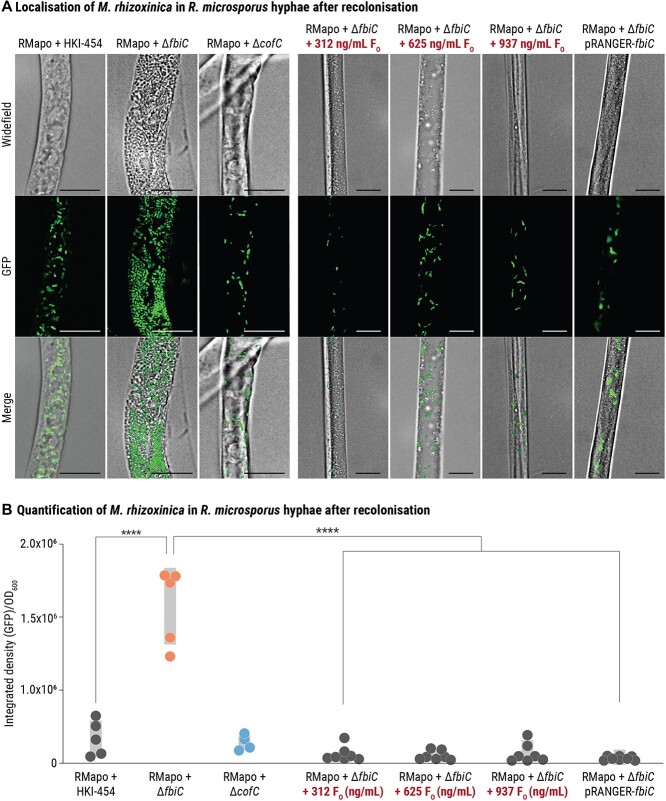
Superresolution imaging and quantification of *M. rhizoxinica* strains inside of mycelia of *R. microsporus*; (A) microscopy images of apo-symbiotic *R. microsporus* (RMapo) recolonized by *M. rhizoxinica* wild type (RMapo + HKI-454), *M. rhizoxinica* Δ*fbiC* (RMapo + *M. rhizoxinica* Δ*fbiC*), *M. rhizoxinica* Δ*cofC* (RMapo + *M. rhizoxinica* Δ*cofC*), *M. rhizoxinica* Δ*fbiC* supplemented with various concentrations of synthetic F_O_ (312 ng/ml, 625 ng/ml, and 937 ng/ml), or complemented *M. rhizoxinica* Δ*fbiC* (RMapo + *M. rhizoxinica* Δ*fbiC* pRANGER-*fbiC*); bacterial cells are labeled with GFP (green); scale bars: 10 μm; (B) following co-cultivation and fluorescence microscopy at 485/498 nm, the integrated density per bacterial density (OD_600_) was calculated for apo-symbiotic *R. microsporus* recolonized by *M. rhizoxinica* wild type, *M. rhizoxinica* Δ*fbiC*, *M. rhizoxinica* Δ*cofC*, *M. rhizoxinica* Δ*fbiC* supplemented with various concentrations of synthetic F_O_ (312 ng/ml, 625 ng/ml, and 937 ng/ml), or *M. rhizoxinica* Δ*fbiC* pRANGER-*fbiC*; strain order as given in Panel A; dots represent biological replicates (*n* ≥ 5), grey bars mark ± one standard error of the mean (SEM); one-way ANOVA with Tukey’s multiple comparison test (^*^^*^^*^^*^P<.0001, Supplementary Table 10) was applied.

A lack of sporulation in F_O_-deficient *R. microsporus*–*M. rhizoxinica* co-cultures could be caused by the inability of *M. rhizoxinica* Δ*fbiC* to recolonize fungal hyphae as has been previously reported for *M. rhizoxinica* that lack a functional type 2 secretion system [25]. To investigate whether *M. rhizoxinica* Δ*fbiC* can enter fungal hyphae, we generated *M. rhizoxinica* strains that express GFP constitutively. Apo-symbiotic *R. microsporus* was co-incubated with GFP-expressing *M. rhizoxinica* wild type, *M. rhizoxinica* Δ*fbiC*, or *M. rhizoxinica* Δ*cofC*. After 1 week of co-cultivation, superresolution fluorescence microscopy was performed to visualize individual bacterial cells and fungal hyphae. As expected, wild-type *M. rhizoxinica* is located inside fungal hyphae. *M. rhizoxinica* Δ*fbiC* and *M. rhizoxinica* Δ*cofC* are also clearly visible inside *R. microsporus* hyphae confirming successful fungal recolonization by both of these strains (Fig. 6A). Fungal mycelium recolonized by *M. rhizoxinica* Δ*fbiC* displays a different morphology, both at the macroscopic and microscopic scale, when compared to that recolonized by *M. rhizoxinica* wild type and *M. rhizoxinica* Δ*cofC* (Figs 4A and 6A). When grown on agar plates, the mycelium containing *M. rhizoxinica* Δ*fbiC* grows flat and patchy in contrast to the fluffy, aerial mycelium of the wild type and *M. rhizoxinica* Δ*cofC*. A combination of widefield and fluorescence microscopy revealed hyphae with a larger diameter (Fig. 6A) containing a significantly higher number of bacterial cells (*P <* .0001) than hyphae containing *M. rhizoxinica* wild type or *M. rhizoxinica* Δ*cofC* (Fig. 6B and Supplementary Table 10).

The high bacterial load of *M. rhizoxinica* Δ*fbiC* in fungal hyphae is reversible through the addition of synthetic F_O_. By adding increasing concentrations of synthetic F_O_ (312 ng/ml, 625 ng/ml, or 937 ng/ml) to apo-symbiotic *R. microsporus*–*M. rhizoxinica* Δ*fbiC* co-cultures, the bacterial load of *M. rhizoxinica* Δ*fbiC* inside the fungal hyphae is comparable to the wild type in all cases (Fig. 6A and Fig. 6B). Quantification of *M. rhizoxinica* Δ*fbiC* pRANGER-*fbiC* (complemented *M. rhizoxinica* Δ*fbiC*) reveals significantly fewer countable bacterial cells inside fungal hyphae compared to *M. rhizoxinica* Δ*fbiC* (*P <*.0001, Fig. 6B and Supplementary Table 10). The ability of F_O_-deficient *M. rhizoxinica* to efficiently recolonize their fungal host combined with the abolishment of fungal sporulation shows that F_O_ is not essential for host colonization but rather supports a process that triggers host sporulation in this bacterial–fungal symbiosis.

## Discussion

The specialized redox cofactor F_420_ is instrumental for diverse biochemical processes in archaea and *Actinobacteria*, e.g. biosynthesis of antibiotics [39], methanogenesis [17], or the degradation of chemical pollutants [40]. In recent years, F_420_ has gained considerable interest due to its low redox potential, which allows for F_420_-dependent enzymes to be utilized in biotechnological applications, such as bioremediation and biosynthetic production of antibiotics [41–44]. This has invigorated a search for new F_420_-dependent enzymes leading to the discovery of F_420_ biosynthetic gene clusters in a wide range of Gram-negative bacteria. We have previously shown that the Gram-negative bacterium *M. rhizoxinica* HKI-454 (Class: *Betaproteobacteria*) is able to produce an unusual derivative of F_420_ (3PG-F_420_) [12]. However, virtually nothing was known about the role of the products of the F_420_ pathway in *M. rhizoxinica* and in Gram-negative bacteria in general.

**Figure 7:**
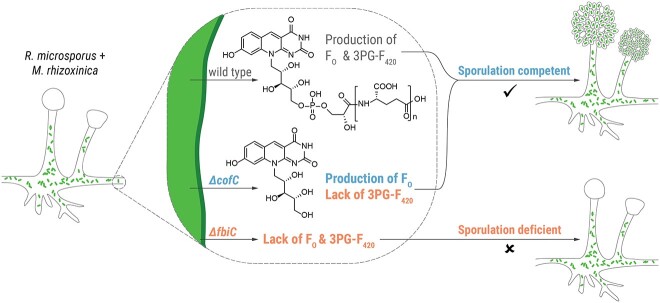
Schematic model of F_O_-dependent host control by endofungal bacteria; the recolonization of apo-symbiotic *R. microsporus* by the wild-type endosymbiont (*M. rhizoxinica* wild type) restores the host’s ability to sporulate; *M. rhizoxinica* that is able to produce F_O_ but lacks the cofactor 3PG-F_420_ (*M. rhizoxinica* Δ*cofC*) do not diminish the sporulation ability of *R. microsporus*; in contrast, *M. rhizoxinica* incapable of producing 3PG-F_420_ and F_O_ (*M. rhizoxinica* Δ*fbiC*) does not trigger sporulation of *R. microsporus*.

Here, we show that the genetic inventory to produce cofactor 3PG-F_420_, although rather rare in related bacteria, is conserved in the genomes of endofungal *Mycetohabitans* strains that are known to form a stable symbiosis with their corresponding *R. microsporus* hosts [27]. Gene expression analysis and metabolic profiling showed that biosynthesis of 3PG-F_420_ and its precursor F_O_ are particularly pronounced during symbiotic growth. By establishing a CRISPR-assisted cytidine base editing strategy for *M. rhizoxinica*, we were able to generate *M. rhizoxinica* strains deficient of F_O_ and 3PG-F_420_ (*M. rhizoxinica* Δ*fbiC*) and deficient of only 3PG-F_420_ (*M. rhizoxinica* Δ*cofC*). Our CRISPR-based, markerless gene inactivation method represents an improvement over previous methods based on homologous recombination and counter-selectable markers [24] as it avoids possible polar effects. Co-culture experiments unequivocally demonstrate that sporulation of the host is only triggered by bacteria that produce F_O_ or when supplemented with F_O_. *M. rhizoxinica* Δ*cofC*, which produces F_O_ but not 3PG-F_420_, does not show any obvious effect on sporulation (Fig. 7). These results demonstrate that F_O_ is a crucial symbiosis factor that controls fungal reproduction.

To probe whether F_O_ is required by *M. rhizoxinica* itself, we tested if F_O_ deficiency negatively impacts bacterial growth *in vitro* but did not observe any growth defects of *M. rhizoxinica* Δ*fbiC*, at least in axenic culture. *In vivo*, we even observed an increased bacterial cell density of *M. rhizoxinica* Δ*fbiC* in *R. microsporus* hyphae. Thus, it is unlikely that F_O_ deficiency causes growth impairments or insufficient protective responses under symbiotic conditions, as such traits would result in poor host colonization and reduced intracellular survival of *M. rhizoxinica* [23]. In fact, an increase in *M. rhizoxinica* Δ*fbiC* cell density following fungal recolonization mirrors an unusual phenomenon recently reported in *R. microsporus* reinfected with *M. rhizoxinica* lacking *Mycetohabitans* transcription activator-like effector 1 (MTAL1) [23]. Based on microfluidics and fluorescence microscopy, it was shown that MTAL1-deficient *M. rhizoxinica* are trapped in hyphae through septa formation leading to high cell numbers. Although we did not observe septa formation and subsequent trapping of *M. rhizoxinica* in this study, it is of course possible that F_O_ deficiency causes a similar response by the fungus. Alternatively, F_O_ may regulate bacterial cell numbers *in vivo* as a type of quorum sensing molecule. The process of how bacterial numbers are controlled inside hyphae is still unknown as *M. rhizoxinica* does not produce any of the “classical” quorum sensing molecules [11]. Another possible explanation for the high bacterial load observed in this study is an accumulation of bacterial cells due to the lack of sporulation because wild-type bacteria would normally be packed into spores [7]. In line with this hypothesis, *R. microsporus* recolonized by *M. rhizoxinica* Δ*cofC* shows a normal bacterial load and produces a similar number of spores as the wild type. Because *M. rhizoxinica* supplies its fungal host with selected amino acids [11], it is conceivable that a bacterial nutrient that is abundant during vegetative growth is consumed by the fungus once the sporulation starts, thus becoming a growth limiting factor. The higher cell density might consequently not be directly related to altered metabolic capacities of the bacteria but rather to an altered metabolism of the fungus.

As symbionts often deliver essential metabolites like vitamins or amino acids to their host [45, 46], F_O_ might be secreted into the host cytosol and subsequently utilized by the fungus. Indeed, F_O_ is present in the supernatant of *M. rhizoxinica* [12, 47] and might, therefore, act as a secreted “vitamin” or “signal” that triggers host sporulation. In support of this hypothesis, *M. rhizoxinica* is not actively engulfed by *R. microsporus* during entry into hyphae and is thus lacking a fungal cell membrane around the bacteria [25]. In addition, F_O_ represents the deazaflavin “head” moiety of 3PG-F_420_, which is redox active [48] and capable of harvesting light [13]. The sporulation-triggering activity of F_O_ could involve its photochemical properties [13]. The light-harvesting properties of F_O_ are exploited by some DNA photolyases, which are highly efficient DNA repair enzymes [49]. Because DNA photolyases have a high binding-specificity to DNA, one could imagine a possible activation or repression of fungal genes in a F_O_-dependent manner. However, future studies will have to rely on biochemical methods such as chemical proteomics to reveal binding partners of F_O_ within the endofungal proteome.

Intrigued by the complete abrogation of fungal sporulation in *R. microsporus* recolonized by *M. rhizoxinica* Δ*fbiC*, we investigated whether F_O_ alone can trigger *R. microsporus* sporulation and observed that the presence of *M. rhizoxinica* is a prerequisite for host sporulation. This observation is in line with previous studies suggesting that control of fungal sporulation involves a multitude of bacterially produced symbiosis factors such as transcription activator-like effectors [9], the secondary metabolite habitasporin [10], or a specialized lipopeptide O-antigen [8]. It was shown that a lack of these molecules reduces the sporulation ability of *R. microsporus* when recolonized by *M. rhizoxinica.* Here, we report complete abrogation of sporulation after host colonization by *M. rhizoxinica* Δ*fbiC*. Such a complete absence of sporulation was previously only reported for *M. rhizoxinica* strains unable to recolonize the apo-symbiotic host due to the absence of intact type 2 [25] or type 3 secretion systems [24]. Our results demonstrate that F_O_, the precursor of 3PG-F_420_, is yet another symbiosis factor that is instrumental for the maintenance of the *R*. *microsporus*–*M*. *rhizoxinica* alliance.

The purpose of 3PG-F_420_ production in *M. rhizoxinica* remains mysterious, as *M. rhizoxinica* Δ*cofC*, which produces F_O_ but not 3PG-F_420_, does not show any obvious effect on fungal sporulation or recolonization. It has been shown that 3PG-F_420_ is typically concentrated in bacterial cell pellets [12, 47], indicating that 3PG-F_420_ may still play as-yet-unknown physiological roles in *Mycetohabitans* species. It is, however, questionable whether 3PG-F_420_ actually acts as a cofactor in *Mycetohabitans* species, as bioinformatics-based evidence for the presence of genes encoding putative F_420_-dependent oxidoreductases in *M. rhizoxinica* could not be obtained [12]. This finding speaks against a cofactor role, because F_420_-dependent enzymes typically belong to well-studied enzyme families and at least two such enzymes would be necessary to drive F_420_-dependent redox biochemistry [20, 21]. It is, however, still possible that uncharacterized enzymes catalyze the 3PG-F_420_-dependent reactions. Alternatively, the lack of F_420_-dependent oxidoreductases could potentially be explained by 3PG-F_420_ fulfilling a non-cofactor role in *M. rhizoxinica*, as has been reported for the cofactor nicotinamide adenine dinucleotide [50, 51] and for the F_420_-derivative factor F_390_ in *Methanothermobacter thermautotrophicus* [52–54].

In this study, we combined targeted gene inactivation, metabolic profiling, colonization assays, and microscopy to functionally characterize deazaflavins of the endofungal symbiont *M. rhizoxinica*. We found that F_O_, the redox-active moiety of F_420_, is necessary for sporulation of the fungal host, signifying that F_O_ is an essential symbiosis factor for the *Rhizopus*–*Mycetohabitans* symbiosis (Fig. 7). This study represents an unprecedented case of a deazaflavin metabolite controlling the reproduction of a eukaryotic host. It seems plausible that F_O_ acts as a mediator of fungal sporulation, but the molecular details of how this non-cofactor role is implemented remain to be deciphered. It should be mentioned that addition of the tail moiety to F_O_, to form the ultimate product of the biosynthetic pathway, may direct 3PG-F_420_ toward possibly acting as a classical cofactor within *M. rhizoxinica*. Although the role of atypical metabolites in symbioses and microbial communities in general has been hitherto overlooked, this study showcases the remarkable flexibility of biochemical molecules derived from a relatively simple pathway. Our results should inspire future research avenues into the functional elucidation of F_420_ pathway products involved in cellular communication and symbiosis.

## Supplementary Material

SI_F420_240423_wrae074

## Data Availability

All data is included in this manuscript.
