# Supplementary material for: Deazaflavin metabolite produced by endosymbiotic bacteria controls fungal host reproduction
Source: ISME J. 2024 May 1;18(1):wrae074. doi: 10.1093/ismejo/wrae074 (PMC11104420; doi:10.1093/ismejo/wrae074)
Supplement: SI_F420_240423_wrae074 [file si_f420_240423_wrae074.docx]

Supplementary Information for

**Deazaflavin metabolite produced by endosymbiotic bacteria controls fungal host reproduction**

Ingrid Richter^1,^*, Mahmudul Hasan^2,#^, Johannes W. Kramer^1,#^, Philipp Wein^1^, Jana Krabbe^1^, Philip Woitas^3^, Timothy P. Stinear^4^, Sacha J. Pidot^4^, Florian Kloss^3^, Christian Hertweck^1,5,6^, Gerald Lackner^2,7^*

*Co-corresponding authors: Gerald Lackner, [Gerald.Lackner@uni-bayreuth.de](mailto:Gerald.Lackner@uni-bayreuth.de); Ingrid Richter, [Ingrid.Richter@leibniz-hki.de](mailto:Ingrid.Richter@leibniz-hki.de)

^#^Contributed equally to this work.

**This PDF file includes:**

Materials and Methods

Supplementary Figures 1 to 6

Supplementary Tables 1 to 10

SI References

**Materials and Methods:**

## RNA extraction from axenic and symbiotic *M. rhizoxinica*

For RNA extraction from axenic *M. rhizoxinica* HKI-454, bacteria were grown in 2 mL MGY M9 minimal medium at 30°C and 110 rpm. After five days of incubation, cells (2 mL) were centrifuged and RNA was extracted from the cell pellet using the Quick-RNA Fungal/Bacterial Miniprep Kit (Zymo Research, Irvine, CA, USA) following the manufacturers’ recommendations. Because there is no standard method for RNA extraction from endofungal bacteria, we developed our own protocol for reliable RNA extraction from symbiotic *M. rhizoxinica* (**Supplementary Fig. 2a**). Wild-type *R. microsporus* was grown on 15 large (145 mm diameter) PDA petri dishes (containing 2% agar) at 30°C for five days or until the fungal mycelium covers in the entire plate surface. The fungal mycelium was harvested from all 15 plates and collected in a 500 mL sterile Schott bottle. Sterile water (300 mL) was added to the mycelium before homogenization using an Ultra Turrax® (IKA Labortechnik, Staufen, Germany). The homogenized mycelium was filtered through a 40 µm cell strainer (Corning Inc., Corning, NY, USA) followed by filtration through a 5 µm Acrodisc® syringe filter (Cytiva, Marlborough, MA, USA). The flow-through (containing endobacteria) was centrifuged at 8,000 x *g* for 5 min at 4°C and the supernatant was discarded. For RNA extraction, the cell pellet (containing endobacteria) was resuspended in 2 mL TRIzol reagent (Invitrogen, Waltham, MA, USA) and immediately frozen over liquid nitrogen. The sample was thawed on ice, and incubated at room temperature for 5 min followed by the addition of 200 µL chloroform. The sample was vortexed for 15 s and then centrifuged at 10,000 x *g* for 5 min at 4°C. The aqueous, upper phase (containing RNA) was transferred to a fresh 2 mL safe locker tube without disturbing the interphase. 200 µL UltraPure phenol:chloroform:isoamyl alcohol (25:24:1, v/v, Invitrogen) was added to the RNA-containing phase, followed by vortexing for 15 s and centrifugation at 10,000 x *g* for 5 min at 4°C. The aqueous, upper phase (containing RNA) was again transferred to a fresh 2 mL safe locker tube, 200 µL UltraPure phenol:chloroform:isoamyl alcohol (Invitrogen) was added followed by vortexing and centrifugation. These steps were repeated until the absence of a white interphase. When no interphase was visible, the aqueous, upper phase (containing RNA) was not transferred to a fresh 2 mL safe locker tube but instead 500 µL chloroform was directly added to the tube containing the the aqueous, upper phase and phenol:chloroform:isoamyl alcohol lower phase. The sample was vortexed for 15 s and centrifuged at 10,000 x *g* for 5 min at 4°C. The aqueous, upper phase (containing RNA) was transferred to a fresh 2 mL safe locker tube. To precipitate RNA, 200 µL cold isopropanol was added, the tube was inverted several times and then incubated overnight -20°C. The next day, the samples were centrifuged at 10,000 x *g* for 1 h at 4°C and the supernatant was discarded. The cell pellet was washed three times with 700 µL 70% ethanol before air-drying at 37°C until the pellet became transparent. The dry pellet was redissolved in 30 µL RNase-free water and stored at -80°C.

To remove genomic DNA, 30 µL RNA aliquots (extracted from symbiotic or axenic *M. rhizoxinica*) were digested with TURBO DNase (Invitrogen) following the manufacturer’s recommendations. Following the DNase digest, samples were cleaned using the RNA Clean & Concentrator Kit (Zymo Research) following the manufacturer’s recommendations. RNA quality was confirmed by running a 1% agarose gel and RNA concentrations were measured using a NanoDrop (Thermo Scientific, Waltham, MA, USA).

## cDNA synthesis and DNase success check

First-strand cDNAs were synthesized from total RNA (1040 ng) using random hexamer primers supplied with the Transcriptor First Strand cDNA Synthesis Kit (Roche Diagnostics, Penzberg, Germany). Negative control reactions in which sterile water replaced the reverse transcriptase (-RT) were carried out in parallel. To confirm that no gDNA is present in the final cDNA samples, we performed end-point PCR using primers (**Supplementary Table 2a**) targeting the gene *rpoB*, which encodes the β subunit of the bacterial RNA polymerase. PCRs were carried out in 20 µL final volumes containing: 10 µL One*Taq*® Quick-Load® 2X Master Mix (New England Biolabs, Ipswich, MA, USA), forward and reverse primers (both 0.2 µM), 6 µL dH_2_O, and 2 µL template cDNA. As template cDNA, cDNA reaction mixtures were used that contain reverse transcriptase (+RT) and cDNA reaction mixtures that lack reverse transcriptase (-RT). The following thermocycling conditions were used: 96°C/3 min, 1 cycle; 96°C/10 s, 60°C/15 s, 68°C/30 s, 30 cycles; 68°C/5 min, 1 cycle; 16°C/hold. The resulting PCR products were visualized on an ethidium bromide-stained 2% agarose gel. A lack of amplification in -RT cDNA samples confirmed that gDNA was completed digested during RNA extractions (**Supplementary Fig. 2d**).

## LC-MS analysis

LC-MS measurements were performed using a Dionex Ultimate 3000 UHPLC (Thermo Scientific) and a reversed phase C18 column (Luna Omega 1.6 µm, 100 Å, 100 x 2.1 mm, Phenomenex, Torrance, CA, USA) coupled with a QExactive Plus Orbitrap mass spectrometer (Thermo Scientific). After injection of 5 µL of extracts (see above), chromatography was performed at 40°C using water (A) and acetonitrile (B), both acidified with formic acid 0.1%, for gradient elution (0 min, 5% B; 1 min, 15% B; 3 min, 25% B; 6 min, 40% B; 7–9 min, 97% B; 10–13 min, 5% B). Full MS measurements were made in positive mode at a resolution of 70,000 and within the 200–2000 m/z range. For the tandem mass spectrometry (MS/MS), the top ten simultaneous peaks were selected for fragmentation. The resolution was reduced to 17.500 and stepped collision energies of 20, 30, and 40 eV (normalized to 500 m/z) were applied.

## Generation of the base editing plasmids pTsK-AnCU-fbiC (targeting *fbiC*) and pTsK-AnCU-cofC (targeting *cofC*).

The plasmid pTsK-CasRed-Bt was digested with *Kpn*I and *Mlu*I (New England Biolabs) to remove the Red-operon as well as the 33 bp of the *cas9** gene. Using gene synthesis (Twist Bioscience, South San Francisco, CA, USA) we obtained a gene cassette consisting of a codon-optimized gene for the rat cytidine deaminase rAPOBEC1 (1) that is under the control of the rhamnose inducible *rhaB* promoter and fused it to the 5’ end of the *cas9** gene over a XTEN-linker (1). This way, a point mutation (D10A) was introduced into *cas9** that changes the enzyme activity of the Cas9 from a double to a single strand break (nickase nCas9). This synthetic construct was received from its subcloning vector by *Kpn*I/*Mlu*I (New England Biolabs) digest and was then ligated (T4 DNA ligase, New England Biolabs) with the enzyme-digested pTsK-CasRed-Bt plasmid resulting in the new plasmid pTsK-AnC (**Supplementary Table 4**), which contains the rAPOBEC1-XTEN-nCas9 cassette.

To enhance the base editing efficiency, the rAPOBEC1-XTEN-nCas9 cassette was fused with the codon-optimized gene for the Uracil DNA glycosylase inhibitor (UGI) (1). For this purpose, we designed a subcloning vector containing the final 151 bp of *cas9** that was fused to *ugi** over a short spacer (SGGS) by gene synthesis. In addition, the synthetic DNA sequence also contains the sgRNA cassette. This cassette consists of the constitutive J23119 promoter (2) and an unspecific spacer N20 sequence harboring an *Afe*I and a *Mfe*I site for easy adaptation of the N20 sequence to the target. Both plasmids, the synthetic subcloning vector as well as pTsK-AnC, were digested with *Psp*OMI/*Asc*I (New England Biolabs) and ligated (T4 DNA ligase, New England Biolabs) resulting in the general base editing plasmid pTsK-AnCU-sg (**Supplementary Table 4**). The editing window of this construct is within the range of –13 to –17 counting the PAM as position 1–3 (3).

Possible target sites for base editing that would result in a stop codon were identified either manually or using CRISPy-web (4). To exchange the spacer N20 sequence, pTsK-AnCU-sg was digested with *Afe*I/*Mfe*I (New England Biolabs) and assembled with the specified N20 sequence using NEBuilder (New England Biolabs). The individualized 60 bp inserts were obtained by primer self-annealing. The primer pair JK941/JK942 (**Supplementary Table 2b**) was used to generate pTsK-AnCU-fbiC (**Supplementary Table 4**) targeting the *fbiC* gene and the *cofC* knockout plasmid pTsK-AnCU-cofC (**Supplementary Table 4**) was obtained using the primer pair JK945/JK946 (**Supplementary Table 2b**). To reduce false positive clones during plasmid construction, the assembly reaction was digested with *Mfe*I (New England Biolabs) prior to its transfer into *E. coli* TOP10 by electroporation.

The base editing plasmids pTsK-AnCU-fbiC and pTsK-AnCU-cofC were used to transform competent *M. rhizoxinica* by electroporation. Briefly, wild-type *M. rhizoxinica* were grown in 20 mL MGY medium at 30°C and orbital shaking until an OD_600_ of approximately 0.6. The cells were harvested by centrifugation at 6,000 x *g* for 5 min and room temperature. The cell pellet was washed three times with 20 mL 300 mM sucrose and then resuspended in 1 mL 300 mM sucrose. The plasmid (2 µL) was added to 60 µL of cells prior to electroporation at 25 µF, 200 Ω and 2.5-3 kV (Eppendorf Eporator®, Eppendorf, Hamburg, Germany). The transformants were incubated in 1 mL MGY at 30°C for 4–6 h with orbital shaking to recover. Subsequently, the cells were harvested by centrifugation at 6,000 × *g* for 2 min and resuspended in approximately 100 µL MGY. The cell suspension was spread onto NAG petri dishes supplemented with 50 µg/mL kanamycin and incubated at 30°C until colonies appeared. Single colonies were picked, then streaked onto kanamycin-containing NAG petri dishes that were previously treated with 50 µL l-rhamnose (100 g/L) solution. After incubation at 30°C, colonies were screened for the desired mutations by amplifying a portion of the target genes with colony PCR using the oligonucleotide primer pairs JK953/JK954 (for *fbiC*) or JK957/JK958 (for *cofC*) followed by Sanger sequencing (Eurofins Genomics, Ebersberg, Germany, **Supplementary Table 2b**). Colonies containing a mixture of mutant and wild type were re-streaked onto NAG petri dishes (containing kanamycin and l-rhamnose) until pure clones of *M. rhizoxinica* Δ*fbiC* and *M. rhizoxinica* Δ*cofC* were obtained. In order to remove the base editing plasmids, the mutants were cultured overnight in MGY (without supplements) at 37°C with orbital shaking.

## Genome sequencing of *M. rhizoxinica* Δ*fbiC* and *M. rhizoxinica* Δ*cofC*

Genomic DNA (gDNA) was isolated from axenic *M. rhizoxinica* Δ*fbiC* and *M. rhizoxinica* Δ*cofC* using the MasterPure DNA Purification Kit (Biozym Scientific, Hessisch Oldendorf, Germany) following the manufacturers’ recommendations. The gDNA was quantified using a NanoDrop (Thermo Scientific) and used for shotgun sequencing. Sequencing was performed using the Nextera XT Library preparation kit in combination with a NextSeq System (Illumina, San Diego, CA, USA) (5) producing paired-end reads with a mean length of approximately 150 base pairs.

Raw sequence reads were trimmed with trimmomatic (6) and reference assemblies were performed using Spades v3.7.0, with the previously published *M. rhizoxinica* HKI-454 genome (7) as a reference. Variants were called by aligning the sequenced reads of the Δ*fbiC* and Δ*cofC* mutants to the *M. rhizoxinica* HKI-454 reference genome using Snippy v4.4.5 (<https://github.com/tseemann/snippy>). SNP analysis revealed that there were no other deleterious off-target SNPs in the genome of either mutant.

## Generation of genetically complemented *M. rhizoxinica* Δ*fbiC* and *M. rhizoxinica* Δ*cofC*

To genetically complement the mutant strains *M. rhizoxinica* Δ*fbiC* and *M. rhizoxinica* Δ*cofC*, the native promotor binding site of *fbiC* and *cofC* was predicted using the software Prediction of Bacterial Promoters (BPROM) (8). Primers for amplification of *fbiC* and *cofC* (oMH63/oMH64) were designed to include the native promoter binding site (**Supplementary Table 2d**). Both the *fbiC* and *cofC* genes were amplified together with the native promoter binding site in PCR reactions containing boiled *M. rhizoxinica* HKI-454 cells, Q5 High-Fidelity DNA Polymerase (New England Biolabs), and the primer pair oMH63/oMH64 (**Supplementary Table 2d**). The commercial plasmid pRANGER-BTB3®, a broad host range expression vector with pBBR1 replicon, was linearized using the primer pair pRANGER_SpeI_F/pRANGER_NdeI_R (**Supplementary Table 2d**). The purified PCR amplicon was cloned into the linearized pRANGER using the FastCloning protocol (9) yielding pMH41 (**Supplementary Table 4**). The primer pair oMH697oMH70 was used for the amplification of *fbiC* (from pMH41) that led to the construction of pMH71 (pRANGER-*fbiC,* **Supplementary Fig. 5a, Supplementary Table 4** and **Table 2d**). The primer pair oMH112/oMH113 was used for the amplification of *cofC* (from pMH41) that led to the construction of pMH72 (pRANGER-*cofC*, **Supplementary Fig. 5b, Supplementary Table 4** and **Table 2d**). The reaction mixture was subsequently used to transform *E. coli* Top10 (Invitrogen^TM^, One Shot^TM^) via electroporation. Constructed plasmids were confirmed by Sanger sequencing (Eurofins Genomics).

The new plasmids pRANGER-*fbiC* and pRANGER-*cofC* were introduced into competent *M. rhizoxinica* Δ*fbiC* and *M. rhizoxinica* Δ*cofC*, respectively, as described above. Transformants were grown on NAG containing 50 µg/mL chloramphenicol. Colonies containing the respective plasmids were observed using colony PCR and control primers (**Supplementary Fig. 5c** and **Supplementary Table 2d**).

## Assessment of growth and metabolic activity of *M. rhizoxinica* wild type and *M. rhizoxinica* Δ*fbiC* on different carbon sources

Pre-cultures of *M. rhizoxinica* wild type and *M. rhizoxinica* Δ*fbiC* were grown in MGY M9 medium overnight at 30°C and 110 rpm. After one day of incubation, bacterial cells were washed with M9 Salts and then inoculated in 20 mL M9 minimal medium (5 g/L MgSO_4_, M9 salts: 12.8 g/L Na_2_HPO_4_ x 7H_2_O, 3 g/L KH_2_PO_4_, 500 mg/L NaCl, 1 g/L NH_4_Cl; trace elements: 10 mg/L EDTA, 1 mg/L CaCl_2_ x 2H_2_O, 0.2 mg/L Na_2_MoO_4_ x 2H_2_O, 0.4 mg/L CoCl_2_ x 6H_2_O, 1.2 mg/L MnCl_2_ x 4H_2_O, 2 mg/L ZnSO_4_ x 7H_2_O, 5 mg/L FeSO_4_ x 7H_2_O, 0.2 mg/L CuSO_4_ x 5H_2_O; vitamins: 25 µg/L 4-aminobenzoic acid, 2 mg/L d(+)-biotin, 50 µg/L cyanocobalamin, 50 µg/L nicotinic acid, 12.5 µg/L calcium-d(+)-pantothenate, 125 µg/L pyridoxamine dihydrochloride monohydrate, 25 µg/L thiamine chloride hydrochloride, 250 µg/L riboflavin; pH 7.2) in 100 mL baffled Erlenmeyer flasks to a uniform starting OD_600_ of 0.2 and incubated at 30°C and 115 rpm for three days. Each bacterial strain was grown in the presence of a single carbon source (2 g/L glycerol, 2 g/L gluconic acid, 2 g/L serin, 2 g/L glutamic acid, 2 g/L glutamine, or 2 g/L aspartic acid). The cell density was monitored over time using light backscattering recorded by a Cell Growth Quantifier (Aquila Biolabs, Baesweiler, Germany). Raw data were analyzed using the CGQuant 8.2.5 software (Aquila Biolabs). Growth curves were generated using GraphPad Prism 9.5.1 (GraphPad Software).

The Biolog Phenotype MicroArray 1 (MediLoc Laborsysteme GmbH, Minden, Germany) was used to assess the metabolic activity on various carbon sources (10). In short, bacteria were grown to an OD_600_ of 1 in MGY M9 minimal medium, washed three times with phosphate buffer (137 mM NaCl, 2.7 mM KCl, 10 mM Na_2_HPO_4_, 1.8 mM KH_2_PO_4_; pH 7.4) followed by an overnight incubation at 30°C and 110 rpm. The next day, cells were pelleted, resuspended in the inoculation buffer of the Biolog plates, and mixed with the colorless redox dye (mix A). Cell suspensions (100 µL) were dispensed into individual wells to a starting OD_600_ of 0.2 and incubated at 30°C for four days. The formation of a violet color, indicating respiratory metabolic activity, was inspected visually by eye.

## Generation of GFP-labelled *M. rhizoxinica* strains

To visualize the localization of *M. rhizoxinica* strains within the fungal hyphae, a GFP-encoding plasmid was constructed as follows. First, pRANGER-BTB3® was *Hind*III (New England Biolabs) digested and assembled with a P_S12_-*pheS* cassette amplified from pGL42a (11) yielding pBBR-pheS, an intermediate plasmid of no interest for this study. However, pBBR-pheS was cut with *Nde*I and *Afl*II (New England Biolabs) to remove the unnecessary *pheS* as well as the cassette for arabinose induction of the originally containing *araB* promoter. This way only the strong, constitutive, ribosomal S12 promoter (12) was retained in the backbone. Next, *gfp* was amplified from pHKT2 using the primer pair pBBR-GFP_fw/pBBR-GFP_rv (**Supplementary Table 2c**) and assembled with enzyme-digested pBBR-pheS using NEBuilder Mix (New England Biolabs) resulting in pBBR-GFP-cml (**Supplementary Table 4**).

To allow plasmid-based GFP co-expression in complemented *M. rhizoxinica* Δ*fbiC* and *M. rhizoxinica* Δ*cofC,* the chloramphenicol resistance cassette was removed from pBBR-GFP-cml by *Bam*HI and *Sna*BI digest (New England Biolabs) and replaced by a gentamycin resistance cassette amplified from pKD46-Gm (13) with the primer pair JK1071/JK1072 (**Supplementary Table 2c**). Assembly of both fragments using NEBuilder Mix (New England Biolabs) resulted in pBBR-GFP-gm (**Supplementary Table 4**).

Finally, pBBR-GFP-cml (**Supplementary Table 4**) was transferred into wild-type *M. rhizoxinica*, *M. rhizoxinica* Δ*fbiC,* and *M. rhizoxinica* Δ*cofC* via electroporation as described above, whereas *M. rhizoxinica* Δ*fbiC* pRANGER-*fbiC* and *M. rhizoxinica* Δ*cofC* pRANGER-*cofC* received the GFP-encoding plasmid pBBR-GFP-gm (**Supplementary Table 4**) via electroporation. Transformed *M. rhizoxinica* cells were plated on NAG agar plates containing either 50 µg/mL chloramphenicol (wild-type *M. rhizoxinica*, *M. rhizoxinica* Δ*fbiC,* and *M. rhizoxinica* Δ*cofC*) or 20 µg/mL gentamycin and 50 µg/mL chloramphenicol (*M. rhizoxinica* Δ*fbiC* pRANGER-*fbiC* and *M. rhizoxinica* Δ*cofC* pRANGER-*cofC*).

## Chemical synthesis of F_O_

F_O_ was synthesized as described before (14). The obtained synthetic material was purified by preparative reversed-phase HPLC on a Shimadzu system (two LC-20AP pumps) equipped with a fraction collector FRC-10A and detection at 254 nm (UV/VIS SPD-M20A) using a Phenomenex SynergiTM 4 µm Fusion-RP column (C18, 80 Å, 250 mm x 21.2 mm). A linear gradient (10% B for 5 min,10% to 100% B in 10 min, 100% B for 10 min) was employed with A: water + 0,1% TFA and B: acetonitrile as eluents. The fractions containing the product were pooled and concentrated under reduced pressure.

1H-NMR (300 MHz, DMSO-d6): δ = 7.07 (dd, J = 8.72, 1.83 Hz, 1 H), 7.41 (d, J = 1.35 Hz, 1H), 8.05 (d, J = 8.85 Hz, 1H), 8.93 (s, 1H), 11.09 (s, 1H), 11.32 (bs, 1H).

## Quantification of bacterial cells inside of fungal hyphae

Fluorescent wide-field images, containing spatial calibration metadata, were imported to Fiji (15) and the same spatial scale was applied to all images using the global scale function. Prior to image analyses, all images were converted to 16-bit gray-scale, which was propagated to all channels (brightfield and 485/498 nm). The hyphal area and integrated density (ID; product of area and mean grey value) of both channels were measured using the freehand tool and measuring tool implemented in Fiji. Subsequent calculations and data visualization were carried out using MS Excel and GraphPad Prism 9.5.1 (GraphPad Software). To correct for the bacterial cell number that was used for co-culture inoculation, ID values were divided by the OD_600_ of bacterial cells (ID/OD_600_) and then plotted.


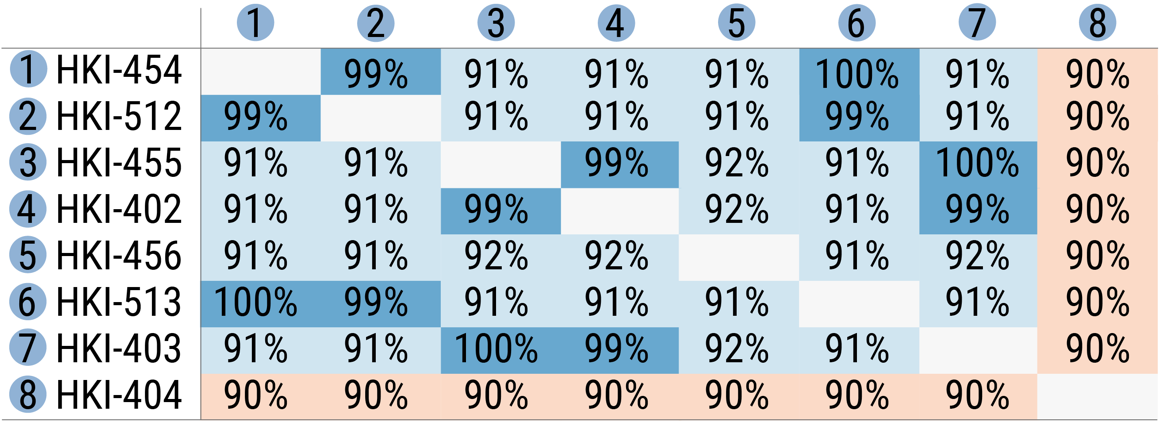


**Supplementary Fig. 1. Identity matrix of 3PG-F_420_-n biosynthetic gene clusters from eight endofungal *Mycetohabitans* species depicting nucleotide identity in percent.** *Mycetohabitans* strain designation: 1: *M. rhizoxinica* HKI-454; 2: *Mycetohabitans* sp. HKI-512; 3: *Mycetohabitans* sp. HKI-455; 4: *Mycetohabitans* sp. HKI-402; 5: *M. endofungorum* sp. HKI-456; 6: *Mycetohabitans* sp. HKI-513; 7: *Mycetohabitans* sp. HKI-403; 8: *Mycetohabitans* sp. HKI-404 (**Supplementary Table 1**).


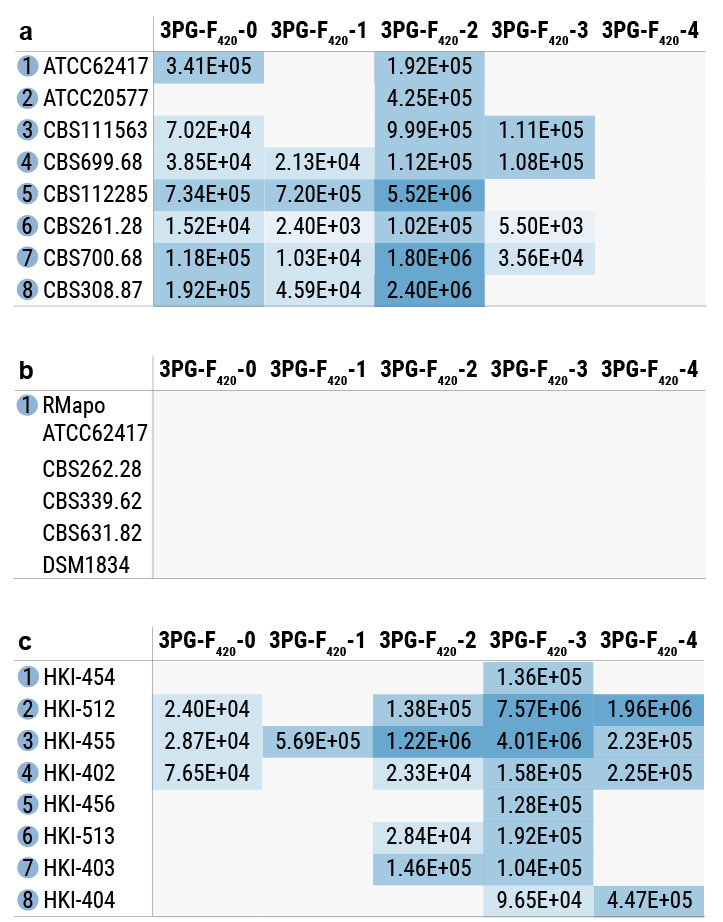


**Supplementary Fig. 2. Detection of 3PG-F_420_-n species using LC-MS/MS.** Heat maps showing normalized abundance of 3PG-F_420_-n species in extracts from (**a**) eight *R. microsporus* strains containing their corresponding *Mycetohabitans* sp. endosymbionts (**Supplementary Table 1**), (**b**) *R. microsporus* (ATCC62417) cured of its endosymbionts (RMapo) and *R. microsporus* strains that are naturally endosymbiont-free, or (**c**) axenic *Mycetohabitans* sp. strains that were isolated from their corresponding *R. microsporus* host. Values represent the area under the curve of extracted ion chromatograms (5 ppm mass tolerance) using the following exact masses ([M+H]^+^): 3PG-F_420_-0: 532.09630, 3PG-F_420_-1: 661.13890, 3PG- F_420_-2: 790.18149, 3PG-F_420_-3: 919.22409, 3PG-F_420_-4: 1048.26668.

**
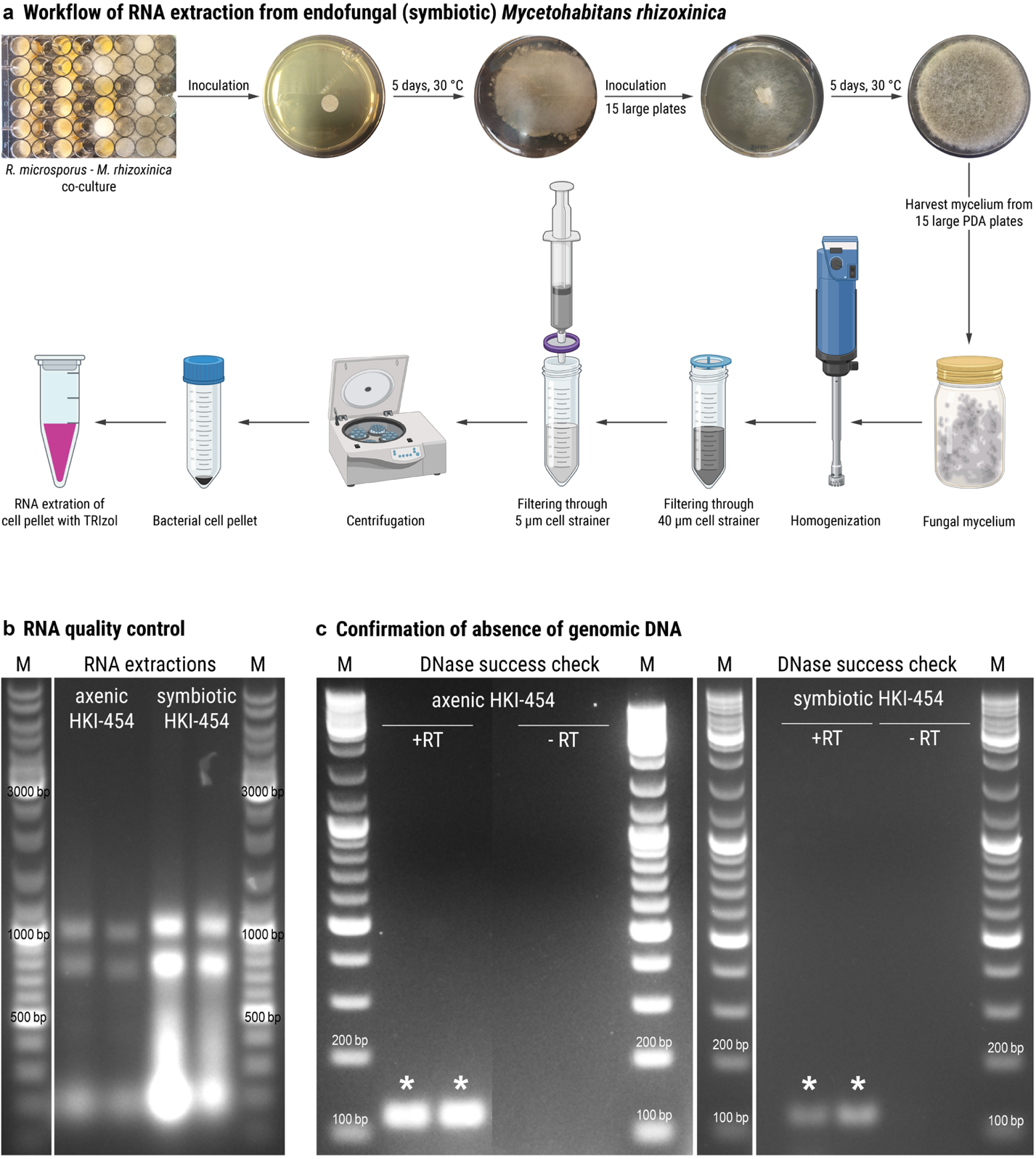
**

**Supplementary Fig. 3. RNA extraction and quality control. a**, Schematic depicting the general workflow for RNA extractions from endofungal (symbiotic) *M. rhizoxinica* wild type (HKI-454). Image created with BioRender. **b**, Confirmation of RNA quality extracted from axenic and symbiotic *M. rhizoxinica* wild type (HKI-454). **c**, Confirmation of absence of genomic DNA in cDNA samples following RNA extraction. Control PCRs of *rpoB* were performed with cDNA reaction mixtures that contain reverse transcriptase (+RT) and cDNA reaction mixtures that lack reverse transcriptase (-RT). PCR products were amplified using *rpoB* primers listed in **Supplementary Table 2a**. Bands corresponding to the expected size are indicated by asterisks (*).


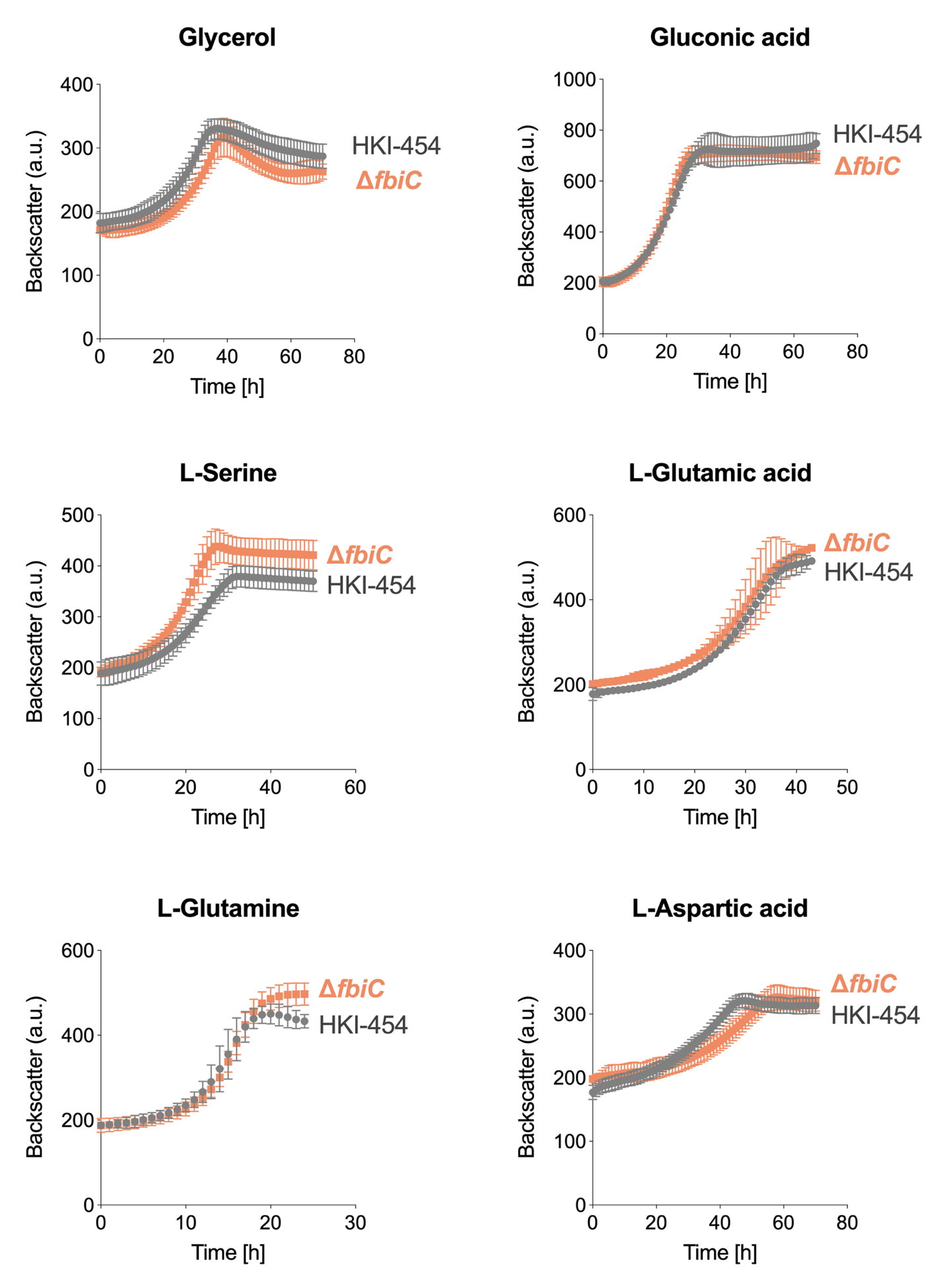


**Supplementary Fig. 4.** **Growth curves of *M. rhizoxinica* wild type (HKI-454) and *M. rhizoxinica* Δ*fbiC.*** Bacterial cultures were grown in M9 minimal medium containing glycerol, gluconic acid, l-serine, glutamine, glutamic acid, or l-aspartic acid as a carbon source in shake flasks. Growth was recorded until cells reached the stationary phase. The solid circles represent an average of measurements from three biological replicates. Error bars represent standard deviations.

**
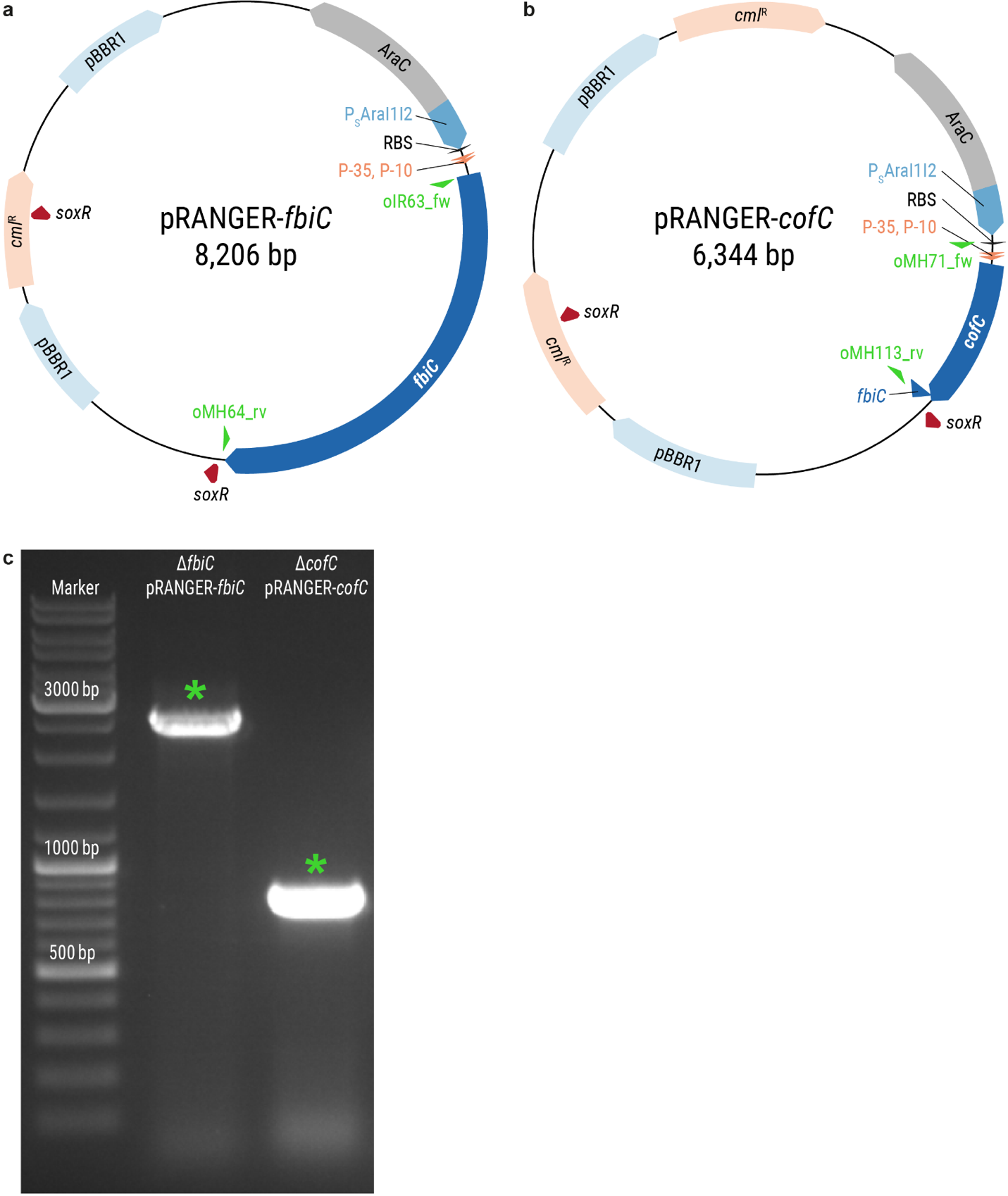
**

**Supplementary Fig. 5.** **Generation and** **confirmation of complemented *M. rhizoxinica* F_420_ knock-out strains.** **a**, Schematic of the plasmid (pRANGER-*fbiC*) used to generate complemented *M. rhizoxinica* Δ*fbiC* strains (*M. rhizoxinica* Δ*fbiC* pRANGER-*fbiC*). **b**, Schematic of the plasmid (pRANGER-*cofC*) used to generate complemented *M. rhizoxinica* Δ*cofC* strains (*M. rhizoxinica* Δ*cofC* pRANGER-*cofC*, **Supplementary Table 4**). **c**, Confirmation of complemented *M. rhizoxinica* Δ*fbiC* (Δ*fbiC* pRANGER-*fbiC*) and complemented *M. rhizoxinica* Δ*cofC* (Δ*cofC* pRANGER-*cofC*) by colony PCR. PCR products were amplified using control primers listed in **Supplementary Table 2d**. Bands corresponding to the expected size are indicated by asterisks (*).

**
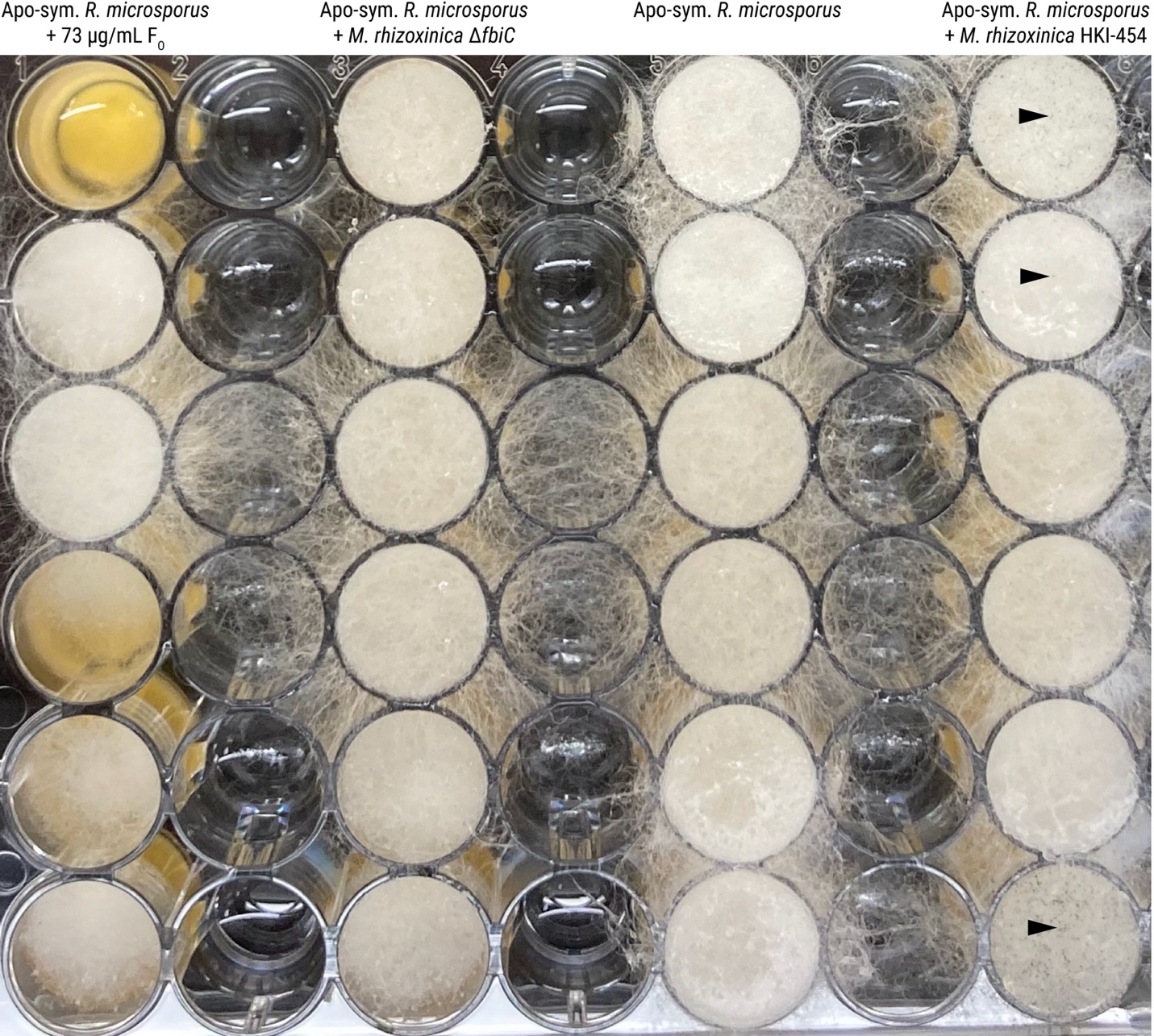
**

**Supplementary Fig. 6. Chemical complementation of apo-symbiotic *R. microsporus* with synthetic F_O_ does not restore the sporulation ability of *R. microsporus.*** Photograph of a co-culture plate containing apo-symbiotic *R. microsporus* (apo-sym) supplemented with 73 μg/mL synthetic F_O_, apo-symbiotic *R. microsporus* co-incubated with *M. rhizoxinica* Δ*fbiC*, apo-symbiotic *R. microsporus*, or apo-symbiotic *R. microsporus* co-incubated with *M. rhizoxinica* wild type (HKI-454). Formation of sporangia is indicated by black arrows.

**Supplementary Table 1.** Fungal strains used in this study that harbor bacterial endosymbionts (16).

| **Taxon** | **Strain designation** | **Origin** | **Bacterial endosymbiont**  **(isolate)** |
| --- | --- | --- | --- |
| *Rhizopus microsporus* van Tieghem | ATCC 62417 | Rice seedlings, Japan | *Mycetohabitans rhizoxinica*  HKI-454 (M1) |
| *Rhizopus* sp. strain F-1360 | ATCC 20577 | Soil, Japan | *Mycetohabitans* sp. strain  HKI-512 (M2) |
| *Rhizopus microsporus* Tieghem var. *microsporus* | CBS 111563 | Sufu starter culture, rice wine tablet, Vietnam | *Mycetohabitans* sp. strain  HKI-455 (M3) |
| *Rhizopus microsporus* Tieghem var. *microsporus* | CBS 699.68 | Soil, Ukraine | *Mycetohabitans* sp. strain  HKI-402 (M4) |
| *Rhizopus microsporus* Tieghem | CBS 112285 | Ground nuts, Mozambique | *Mycetohabitans endofungorum* HKI-456 (M5) |
| *Rhizopus microsporus* var. *chinensis* (Saito) Schipper & Stalpers | CBS 261.28 | Not specified, USA | *Mycetohabitans* sp. strain  HKI-513 (M6) |
| *Rhizopus microsporus* Tieghem var. *microsporus* | CBS 700.68 | Forest soil, Georgia | *Mycetohabitans* sp. strain  HKI-403 (M7) |
| *Rhizopus microsporus* Tieghem var. *microsporus* | CBS 308.87 | Man, from deep necrotic tissue within the hand following a spider bite, Australia | *Mycetohabitans* sp. strain  HKI-404 (M8) |

**Supplementary Table 2. a**, Primers used for gene expression studies. **b**, Primers used for the construction and verification of *M. rhizoxinica* Δ*fbiC* and *M. rhizoxinica* Δ*cofC* mutants. **c**, Primers used for the construction of GFP expressin plasmids. **d**, Primers used for the construction and verification of genetically complemented *M. rhizoxinica* Δ*fbiC* and *M. rhizoxinica* Δ*cofC* mutant strains (*M. rhizoxinica* Δ*fbiC* pRANGER-*fbiC* and *M. rhizoxinica* Δ*cofC* pRANGER-*cofC*).

**a**

| **Gene** | **Name** | **Primer sequence 5’** → **3’** | **Predicted amplicon size (bp)** |
| --- | --- | --- | --- |
| *fbiC* | fbiC_qPCR_fw | CTTGTATCGGTCCTCGTGAAA | 95 |
|  | fbiC_qPCR_rv | CACGATCACTTACGTTGTCAATC |  |
| *cofC* | cofC_qPCR_fw | CTACATCCAACGCCCACTC | 122 |
|  | cofC_qPCR_rv | CCGACCTGGCGCTATTC |  |
| *rpoB* | rpoB_2_Fw | GCTGAAGATGGTCAAGGTGTA | 117 |
|  | rpoB_2_Rv | ATGTCCTCGATCGGAACAATC |  |

**b**

| **Name** | **Sequence 5’** → **3’** (primer binding site) | **Purpose** |
| --- | --- | --- |
| **Plasmid construction** | |  |
| JK941 | gtcctaggtataatgctagc**tggccaagcatttcttaccg**gttttagagctagaaatagc | N20 for  pTsK-AnCU-fbiC |
| JK942 | GCTATTTCTAGCTCTAAAAC**CGGTAAGAAATGCTTGGCCA**GCTAGCATTATACCTAGGAC |  |
| JK945 | gtcctaggtataatgctagc**tccccacaagatctcagcac**gttttagagctagaaatagc | N20 for  pTsK-AnCU-cofC |
| JK946 | GCTATTTCTAGCTCTAAAAC**GTGCTGAGATCTTGTGGGGA**GCTAGCATTATACCTAGGAC |  |
| **Mutant verification** | |  |
| JK953 | gtatgagcttgaagcggtgc | Confirmation of Δ*fbiC* |
| JK954 | GCAGCATCTGGTATTCTGCC |  |
| JK957 | atgtctcctgtttctgtcgc | Confirmation of Δ*cofC* |
| JK958 | CTAGATTTGGCATAGCACCC |  |

**c**

| **Name** | **Sequences 5’** → **3’** | **Purpose** |
| --- | --- | --- |
| pBBR-GFP_fw | CATGAGTCACTAGTGACGAGCCTCAGACTCCAGCGT | Amplification of GFP from pHKT2 (Tomlin *et. al.* 2004) |
| pBBR-GFP_rv | GGAATTCCATATGAGAATCTCCTTCTCTAGCCCAAAAAAACGGGTATGGA |  |
| JK1071 | GGAATTCCATATGAGCCTAAGCCGGGTAATTGTTTC | Amplification of *gm^R^* from pKD46-Gm (Doublet *et. al.* 2008) |
| JK1072 | CATGAGTCACTAGTGTCATTGTTCCGCTTCACTCAAG |  |

**d**

| **Name** | **Sequences 5’** → **3’** | **Purpose** |
| --- | --- | --- |
| pRANGER_SpeI_F | CATGAGTCACTAGTGACGAGCCTCAGACTCCAGCGT | Vector linearization |
| pRANGER_NdeI_R | GGAATTCCATATGAGAATCTCCTTCTCTAGCCCAAAAAAACGGGTATGGA |  |
| oMH63 | GGAATTCCATATGAGCCTAAGCCGGGTAATTGTTTC | Amplification of *fbiC* and *cofC* from gDNA |
| oMH64 | CATGAGTCACTAGTGTCATTGTTCCGCTTCACTCAAG |  |
| oMH69 | ACCATTGCATCTGAGGAATTCGAGTCTTCCATC | Amplification of *fbiC* from pMH41 |
| oMH70 | GAGGAATTCGAGACCATTGCATCTCATATTGTTTC |  |
| oMH112 | TTACAAAATGACATGAGTCACTAGTGACGAGCCTCAGACTCC | Amplification of *cofC* from pMH41 |
| oMH113 | AGTGACTCATGTCATTTTGTAAATAGGATTGCTTACTAGATTTG |  |
| oIR63 | GTGCTATGCCAAATCTAGTAAGC | Confirmation of Δ*fbiC* complementation |
| oMH64 | CTTGAGTGAAGCGGAACAATGA |  |
| oMH71 | CCTAAGCCGGGTAATTGTTTC | Confirmation of Δ*cofC* complementation |
| oMH113 | CAAATCTAGTAAGCAATCCTATTTACAAAATGACATGAGTCACT |  |

**Supplementary Table 3.** Primer efficiencies for qPCR experiments. Primer efficiencies were calculated from standard curves generated with axenic *M. rhizoxinica* cDNA. Properties of qPCR primers are listed in **Supplementary Table 2a**.

| **Gene target** | **Primer pair** | **Slope** | ***R*^2^** | **Y-Inter** | **Efficiency (%)** | **Error** |
| --- | --- | --- | --- | --- | --- | --- |
| *fbiC* | fbiC_qPCR_fw & fbiC_qPCR_rv | –3.47 | 0.99 | 24.13 | 93.9 | 0.054 |
| *cofC* | cofC_qPCR_fw & cofC_qPCR_rv | –3.41 | 0.99 | 20.37 | 96.1 | 0.039 |
| *rpoB* | rpoB_2_fw & rpoB_2_rv | –3.46 | 0.99 | 21.16 | 94.3 | 0.049 |

**Supplementary Table 4.** Plasmids used in this study.

| **Name** | **Description** | **Marker** | **Size** | **Reference** |
| --- | --- | --- | --- | --- |
| **CRISPR/Cas plasmids** | |  |  |  |
| pTsK-CasRed-Bt | Temperature-sensitive shuttle vector for *Burkholderia* (low copy) with L-rhamnose inducible expression of codon-optimized *cas9** and *Burkholderia*-optimized Red-operon for homologous recombination (Redγβα7029) | kan^R^ | 12.1 kb | Niehs *et. al.* 2020 |
| pTsK-AnCU-sg | Temperature-sensitive shuttle vector for *Burkholderia* (low copy) with L-rhamnose inducible expression of codon-optimized fusion protein consisting of rAPOBEC1, nCas9, and UGI, constitutive sgRNA expression | kan^R^ | 11.1 kb | This study |
| pTsK-AnCU-fbiC | CRISPR/Cas base editing vector for *fbiC* knockout | kan^R^ | 11.1 kb | This study |
| pTsK-AnCU-cofC | CRISPR/Cas base editing vector for *cofC* knockout | kan^R^ | 11.1 kb | This study |
| **Plasmids for labelling** | |  |  |  |
| pBBR-GFP-cml | pRANGER-BTB-3 based shuttle vector for *Mycetohabitans* with constitutive GFP expression (ribosomal S12 promoter) | cml^R^ | 3.4 kb | This study |
| pBBR-GFP-gm | pRANGER-BTB-3 based shuttle vector for *Mycetohabitans* with constitutive GFP expression (ribosomal S12 promoter) | gm^R^ | 3.6 kb | This study |
| **Plasmids for genetic complementation** | |  |  |  |
| pMH41 | pRANGER with constitutive expression of *fbiC* and *cofC* | cml^R^ | 8.8 kb | This study |
| pMH71 | pRANGER with constitutive expression of *fbiC* for genetic *trans*-complementation of *M. rhizoxinica* Δ*fbiC* | cml^R^ | 8.2 kb | This study |
| pMH72 | pRANGER with constitutive expression of *cofC* for genetic *trans*-complementation of *M. rhizoxinica* Δ*cofC* | cml^R^ | 6.3 kb | This study |

Abbreveations: kan^R^: kanamycin resistance; cml^R^: chloramphenicol resistance; gm^R^: gentamycine resistance; rAPOBEC1: cytidine deaminase from rat; nCas9: Cas9-nickase with a D10A mutation from *Streptococcus pyogenes*; UGI: DNA glycosylase inhibitor; sgRNA: synthetic guide RNA

**Supplementary Table 5.** Approximate probabilities (*P*) of unpaired *t*-test with Welch’s correction for the relative gene expression of *fbiC* in *M. rhizoxinica* wild type grown in pure, axenic culture (column A) or in symbiosis with their host *R. microsporus* (column B).

| **Unpaired *t*-test with Welch's correction** |  |
| --- | --- |
| *P* value | *0.0455* |
| *P* value summary | * |
| Significantly different (*P<0.05*)? | Yes |
| One- or two-tailed *P* value? | Two-tailed |
| Welch-corrected *t*, df | *t*=2.870, df=4 |
| **How big is the difference?** |  |
| Mean of column A | 1.010 |
| Mean of column B | 1.600 |
| Difference between means (B - A) ± SEM | 0.5900 ± 0.2056 |
| 95% confidence interval | 0.01919 to 1.161 |
| *R*^2^ (eta squared) | 0.67 |
| ***F* test to compare variances** |  |
| *F*, DFn, Dfd | *1.6*, 2, 2 |
| *P* value | *0.7634* |
| *P* value summary | ns |
| Significantly different (*P<0.05*)? | No |

**Supplementary Table 6.** Approximate probabilities (*P*) of unpaired *t*-test with Welch’s correction for the relative gene expression of *cofC* in *M. rhizoxinica* wild type grown in pure, axenic culture (column A) or in symbiosis with their host *R. microsporus* (column B).

| **Unpaired *t*-test with Welch's correction** |  |
| --- | --- |
| *P* value | *0.0135* |
| *P* value summary | * |
| Significantly different (*P<0.05*)? | Yes |
| One- or two-tailed *P* value? | Two-tailed |
| Welch-corrected *t*, df | *t*=4.221, df=4 |
| **How big is the difference?** |  |
| Mean of column A | 1.020 |
| Mean of column B | 4.670 |
| Difference between means (B - A) ± SEM | 3.650 ± 0.8647 |
| 95% confidence interval | 1.249 to 6.051 |
| *R*^2^ (eta squared) | 0.81 |
| ***F* test to compare variances** |  |
| *F*, DFn, Dfd | *41.4*, 2, 2 |
| *P* value | *0.0472* |
| *P* value summary | * |
| Significantly different (*P<0.05*)? | Yes |

**Supplementary Table 7.** Carbon source utilization by *M. rhizoxinica* wild type and *M. rhizoxinica* Δ*fbiC* according to the Biolog Phenotype Microarray system. Plus sign: respiratory activity detected, Minus sign: no activity detectable.

| **Carbon Sources** | ***M. rhizoxinica* wild type** | ***M. rhizoxinica* Δ*fbiC*** |
| --- | --- | --- |
| 1,2-Propanediol | – | – |
| 2-Aminoethanol | – | – |
| 2-Deoxyadenosine | – | – |
| Acetic acid | + | + |
| Acetoacetic acid | – | – |
| Adenosine | – | – |
| Adonitol | – | – |
| Bromo succinic acid | + | + |
| Citric acid | – | – |
| d-Alanine | – | – |
| d-Aspartic acid | + | + |
| d-Fructose | – | - |
| d-Fructose-6-phosphate | – | – |
| d-Galactonic acid-γ-lactone | – | - |
| d-Galactose | – | – |
| d-Galacturonic acid | + | + |
| d-Gluconic acid | + | + |
| d-Glucosaminic acid | – | – |
| d-Glucose-1-phosphate | – | – |
| d-Glucose-6-phosphate | – | - |
| d-Glucuronic acid | – | – |
| d-Malic acid | – | – |
| d-Mannose | – | – |
| d-Psicose | – | – |
| d-Ribose | – | - |
| d-Saccharic acid | – | – |
| d-Serine | – | – |
| d-Sorbitol | – | – |
| d-Threonine | – | – |
| d-Trehalose | – | – |
| d-Xylose | – | – |
| d, l-Malic acid | + | + |
| d, l-α-Glycerol phosphate | – | – |
| d(+)-Melibiose | – | – |
| Dulcitol | – | – |
| Formic acid | + | + |
| Fumaric acid | + | + |
| Glucuronamide | – | – |
| Glycerol | + | + |
| Glycyl-l-aspartic acid | – | – |
| Glycyl-l-proline | – | – |
| l-Alanine | + | + |
| l-Alanyl-glycine | – | – |
| l-Arabinose | – | – |
| l-Asparagine | + | + |
| l-Aspartic acid | + | + |
| l-Fucose | – | – |
| l-Galactonic acid-γ-lactone | – | – |
| l-Glutamic acid | + | + |
| l-Glutamine | + | + |
| l-Lactic acid | – | – |
| l-Lyxose | – | – |
| l-Malic acid | + | + |
| l-Proline | – | – |
| l-Rhamnose | – | - |
| l-Serine | + | + |
| l-Threonine | + | + |
| Lactulose | – | – |
| m-Hydroxy phenyl acetic acid | – | – |
| m-Tartaric acid | – | – |
| Maltose | – | – |
| Maltotriose | – | – |
| Methyl pyruvate | + | + |
| Mono methyl succinate | + | + |
| Mucic acid | – | – |
| myo-Inositol | – | – |
| N-Acetyl-d-glucosamine | – | – |
| N-Acetyl-ß-d-mannosamine | – | – |
| Negative control | – | – |
| p-Hydroxy phenyl acetic acid | – | – |
| Phenylethylamine | – | – |
| Propionic acid | – | – |
| Pyruvic acid | – | – |
| ß-Methyl-d-glucoside | – | – |
| Succinic acid | + | + |
| Sucrose | – | – |
| Thymidine | – | – |
| Tricarballylic acid | – | – |
| Tween 20 | – | - |
| Tween 40 | + | + |
| Tween 80 | + | + |
| Tyramine | – | – |
| Uridine | – | – |
| α-d-Glucose | – | – |
| α-d-Lactose | – | – |
| α-Hydroxy butyric acid | – | – |
| α-Hydroxy glutaric acid-γ-lactone | – | – |
| α-Keto-butyric acid | + | + |
| α-Keto-glutaric acid | – | – |
| α-Methyl-d-galactoside | – | – |

**Supplementary Table 8. a**, Approximate probabilities (P) of Brown-Forsythe test, **b**, one-way analysis of variance (ANOVA), and **c**, Tukey HSD Post Hoc Test for fungal spore counts following co-cultivation of apo-symbiotic R. microsporus (RMapo) with M. rhizoxinica wild type (RMapo + HKI-454), M. rhizoxinica F_420_ mutant strains (RMapo + M. rhizoxinica ΔfbiC and RMapo + M. rhizoxinica ΔcofC), or complemented M. rhizoxinica F_420_ mutant strains (RMapo + M. rhizoxinica ΔfbiC pRANGER-fbiC and RMapo + M. rhizoxinica ΔcofC pRANGER-cofC). Homogeneous data (non-significant Brown-Forsythe) is shown in black numbers. P values with P<0.05 were considered statistically significant (highlighted in grey).

**a**

| **Brown-Forsythe Test** |  |
| --- | --- |
| *F* (DFn, DFd) | *1.8* (5, 34) |
| *P* value | *0.1341* |
| *P* value summary | ns |
| Are SDs significantly different (*P*<*0.05*)? | No |

**b**

| **ANOVA Summary** | |  | | |  |  |  |
| --- | --- | --- | --- | --- | --- | --- | --- |
| *F* | | *3.8* | | |  |  |  |
| *P* value | | *0.0076* | | |  |  |  |
| *P* value summary | | ** | | |  |  |  |
| Significant diff. among means (*P*<*0.05*)? | | Yes | | |  |  |  |
| *R*^2^ | | 0.36 | | |  |  |  |
| **ANOVA Table** | **SS** | | **DF** | **MS** | | ***F* (DFn, DFd)** | ***P* value** |
| Treatment (between columns) | 8.34E+17 | | 5 | 1.67E+17 | | *F* (5, 34) = *3.8* | *0.0076* |
| Residual (within columns) | 1.49E+18 | | 34 | 4.38E+16 | |  |  |
| Total | 2.32E+18 | | 39 |  | |  |  |

**c**

| **Strain Comparison** | | **Mean Diff.** | **95% CI** |  |  | **Summary** | ***P* value** |
| --- | --- | --- | --- | --- | --- | --- | --- |
| RMapo | RMapo + HKI-454 | –3.32E+08 | –6.85E+08 | to | 2.04E+07 | ns | *0.0743* |
|  | RMapo + Δ*fbiC* | –2.31E+06 | –3.55E+08 | to | 3.50E+08 | ns | *>0.9999* |
|  | RMapo + Δ*cofC* | –2.60E+08 | –6.84E+08 | to | 1.64E+08 | ns | *0.45* |
|  | RMapo + Δ*fbiC* pRANGER-*fbiC* | –3.02E+08 | –6.72E+08 | to | 6.87E+07 | ns | *0.1654* |
|  | RMapo + Δ*cofC* pRANGER-*cofC* | –2.81E+08 | –6.64E+08 | to | 1.02E+08 | ns | *0.257* |
| RMapo + HKI-454 | RMapo + Δ*fbiC* | 3.30E+08 | 3.20E+07 | to | 6.28E+08 | * | *0.0229* |
|  | RMapo + Δ*cofC* | 7.26E+07 | –3.07E+08 | to | 4.53E+08 | ns | *0.9919* |
|  | RMapo + Δ*fbiC* pRANGER-*fbiC* | 3.08E+07 | –2.88E+08 | to | 3.49E+08 | ns | *0.9997* |
|  | RMapo + Δ*cofC* pRANGER-*cofC* | 5.12E+07 | –2.82E+08 | to | 3.84E+08 | ns | *0.9971* |
| RMapo + Δ*fbiC* | RMapo + Δ*cofC* | –2.57E+08 | –6.37E+08 | to | 1.23E+08 | ns | *0.3394* |
|  | RMapo + Δ*fbiC* pRANGER-*fbiC* | –2.99E+08 | –6.18E+08 | to | 1.94E+07 | ns | *0.0758* |
|  | RMapo + Δ*cofC* pRANGER-*cofC* | –2.79E+08 | –6.12E+08 | to | 5.44E+07 | ns | *0.1451* |
| RMapo + Δ*cofC* | RMapo + Δ*fbiC* pRANGER-*fbiC* | –4.18E+07 | –4.38E+08 | to | 3.55E+08 | ns | *0.9995* |
|  | RMapo + Δ*cofC* pRANGER-*cofC* | –2.14E+07 | –4.30E+08 | to | 3.87E+08 | ns | *>0.9999* |
| RMapo + Δ*fbiC* pRANGER-*fbiC* | RMapo + Δ*cofC* pRANGER-*cofC* | –2.04E+07 | –3.72E+08 | to | 3.31E+08 | ns | *>0.9999* |

Abbreviations: SS: sum of squares, DF: degrees of freedom, MS: mean square, Mean Diff.: mean difference.

**Supplementary Table 9. a**, Approximate probabilities (P) of Brown-Forsythe test, **b**, one-way analysis of variance (ANOVA), and **c**, Tukey HSD Post Hoc Test for fungal spore counts following co-cultivation of apo-symbiotic R. microsporus (RMapo) with M. rhizoxinica ΔfbiC complemented with synthetic F_O_ (312 ng/mL, 625 ng/mL, or 937 ng/mL). Homogeneous data (non-significant Brown-Forsythe) is shown in black numbers and non-homogeneous data (significant Brown-Forsythe) is highlighted in red numbers. P values with P<0.05 were considered statistically significant (highlighted in grey).

**a**

| **Brown-Forsythe Test** |  |
| --- | --- |
| *F* (DFn, DFd) | *3.9* (3, 16) |
| *P* value | *0.0283* |
| *P* value summary | * |
| Are SDs significantly different (*P*<*0.05*)? | Yes |

**b**

| **ANOVA Summary** | |  | | |  |  |  |
| --- | --- | --- | --- | --- | --- | --- | --- |
| *F* | | *27.4* | | |  |  |  |
| *P* value | | *<0.0001* | | |  |  |  |
| *P* value summary | | **** | | |  |  |  |
| Significant diff. among means (*P*<*0.05*)? | | Yes | | |  |  |  |
| *R*^2^ | | 0.84 | | |  |  |  |
| **ANOVA Table** | **SS** | | **DF** | **MS** | | ***F* (DFn, DFd)** | ***P* value** |
| Treatment (between columns) | 1.34E+14 | | 3 | 4.45E+13 | | *F* (3, 16) = *27.4* | *1.34E+14* |
| Residual (within columns) | 2.59E+13 | | 16 | 1.62E+12 | |  | *2.59E+13* |
| Total | 1.59E+14 | | 19 |  | |  | *1.59E+14* |

**c**

| **Strain Comparison** | | **Mean Diff.** | **95% CI** |  |  | **Summary** | ***P* value** |
| --- | --- | --- | --- | --- | --- | --- | --- |
| RMapo + Δ*fbiC* | RMapo + Δ*fbiC* + 312 ng/mL F_O_ | –3.25E+06 | –5.55E+06 | to | –9.46E+05 | ** | *0.0048* |
|  | RMapo + Δ*fbiC* + 625 ng/mL F_O_ | –4.80E+06 | –7.10E+06 | to | –2.50E+06 | *** | *0.0001* |
|  | RMapo + Δ*fbiC* + 937 ng/mL F_O_ | –7.11E+06 | –9.41E+06 | to | –4.81E+06 | **** | *<0.0001* |
| RMapo + Δ*fbiC*  + 312 ng/mL F_O_ | RMapo + Δ*fbiC* + 625 ng/mL F_O_ | –1.55E+06 | –3.85E+06 | to | 7.54E+05 | ns | *0.2571* |
|  | RMapo + Δ*fbiC* + 937 ng/mL F_O_ | –3.86E+06 | –6.16E+06 | to | –1.56E+06 | ** | *0.001* |
| RMapo + Δ*fbiC*  + 625 ng/mL F_O_ | RMapo + Δ*fbiC* + 937 ng/mL F_O_ | –2.31E+06 | –4.61E+06 | to | –5.95E+03 | * | *0.0493* |

Abbreviations: SS: sum of squares, DF: degrees of freedom, MS: mean square, Mean Diff.: mean difference.

**Supplementary Table 10. a**, Approximate probabilities (P) of Brown-Forsythe test, **b**, one-way analysis of variance (ANOVA), and **c**, Tukey HSD Post Hoc Test for the bacterial load of M. rhizoxinica wild type (HKI-454), M. rhizoxinica ΔfbiC, M. rhizoxinica ΔcofC, M. rhizoxinica ΔfbiC supplemented with various concentrations of synthetic F_O_ (312 ng/mL, 625 ng/mL, and 937 ng/mL), or complemented M. rhizoxinica ΔfbiC (M. rhizoxinica ΔfbiC pRANGER-fbiC) following recolonization of apo-symbiotic R. microsporus. Homogeneous data (non-significant Brown-Forsythe) is shown in black numbers and non-homogeneous data (significant Brown-Forsythe) is highlighted in red numbers. P values with P<0.05 were considered statistically significant (highlighted in grey).

**a**

| **Brown-Forsythe Test** |  |
| --- | --- |
| *F* (DFn, DFd) | *3.3* (6, 37) |
| *P* value | *0.0099* |
| *P* value summary | ** |
| Are SDs significantly different (*P*<*0.05*)? | Yes |

**b**

| **ANOVA Summary** | |  | |  |  |  |
| --- | --- | --- | --- | --- | --- | --- |
| *F* | | *87.4* | |  |  |  |
| *P* value | | *<0.0001* | |  |  |  |
| *P* value summary | | **** | |  |  |  |
| Significant diff. among means (*P*<*0.05*)? | | Yes | |  |  |  |
| *R*^2^ | | 0.93 | |  |  |  |
| **ANOVA Table** | **SS** | | **DF** | **MS** | ***F* (DFn, DFd)** | ***P* value** |
| Treatment (between columns) | 8.39E+12 | | 6 | 1.39E+12 | *F* (6, 37) = *87.4* | *<0.0001* |
| Residual (within columns) | 5.91E+11 | | 37 | 1.60E+10 |  |  |
| Total | 8.98E+12 | | 43 |  |  |  |

**c**

| **Strain Comparison** | | **Mean Diff.** | | **95% CI** |  | |  | | **Summary** | | ***P* value** | |  |
| --- | --- | --- | --- | --- | --- | --- | --- | --- | --- | --- | --- | --- | --- |
| HKI-454 | Δ*fbiC* | | –1.23E+06 | –1.48E+06 | | to | | –9.86E+05 | | **** | | *<0.0001* | |
|  | Δ*cofC* | | 2.81E+04 | –2.36E+05 | | to | | 2.93E+05 | | ns | | *0.9999* | |
|  | Δ*fbiC* + 312 ng/mL F_O_ | | 1.67E+05 | –5.79E+04 | | to | | 3.91E+05 | | ns | | *0.2652* | |
|  | Δ*fbiC* + 625 ng/mL F_O_ | | 1.72E+05 | –5.92E+04 | | to | | 4.02E+05 | | ns | | *0.2633* | |
|  | Δ*fbiC* + 937 ng/mL F_O_ | | 1.56E+05 | –7.51E+04 | | to | | 3.86E+05 | | ns | | *0.3726* | |
|  | Δ*fbiC* pRANGER-*fbiC* | | 1.58E+05 | –6.71E+04 | | to | | 3.82E+05 | | ns | | *0.3279* | |
| Δ*fbiC* | Δ*cofC* | | 1.26E+06 | 9.99E+05 | | to | | 1.53E+06 | | **** | | *<0.0001* | |
|  | Δ*fbiC* + 312 ng/mL F_O_ | | 1.40E+06 | 1.18E+06 | | to | | 1.63E+06 | | **** | | *<0.0001* | |
|  | Δ*fbiC* + 625 ng/mL F_O_ | | 1.41E+06 | 1.18E+06 | | to | | 1.64E+06 | | **** | | *<0.0001* | |
|  | Δ*fbiC* + 937 ng/mL F_O_ | | 1.39E+06 | 1.16E+06 | | to | | 1.62E+06 | | **** | | *<0.0001* | |
|  | Δ*fbiC* pRANGER-*fbiC* | | 1.39E+06 | 1.17E+06 | | to | | 1.62E+06 | | **** | | *<0.0001* | |
| Δ*cofC* | Δ*fbiC* + 312 ng/mL F_O_ | | 1.39E+05 | –1.03E+05 | | to | | 3.80E+05 | | ns | | *0.5629* | |
|  | Δ*fbiC* + 625 ng/mL F_O_ | | 1.43E+05 | –1.04E+05 | | to | | 3.90E+05 | | ns | | *0.5503* | |
|  | Δ*fbiC* + 937 ng/mL F_O_ | | 1.28E+05 | –1.19E+05 | | to | | 3.75E+05 | | ns | | *0.6776* | |
|  | Δ*fbiC* pRANGER-*fbiC* | | 1.29E+05 | –1.12E+05 | | to | | 3.71E+05 | | ns | | *0.6389* | |
| Δ*fbiC* + 312 ng/mL F_O_ | Δ*fbiC* + 625 ng/mL F_O_ | | 4.83E+03 | –1.99E+05 | | to | | 2.09E+05 | | ns | | *>0.9999* | |
|  | Δ*fbiC* + 937 ng/mL F_O_ | | –1.11E+04 | –2.15E+05 | | to | | 1.93E+05 | | ns | | *>0.9999* | |
|  | Δ*fbiC* pRANGER-*fbiC* | | –9.23E+03 | –2.06E+05 | | to | | 1.88E+05 | | ns | | *>0.9999* | |
| Δ*fbiC* + 625 ng/mL F_O_ | Δ*fbiC* + 937 ng/mL F_O_ | | –1.59E+04 | –2.27E+05 | | to | | 1.95E+05 | | ns | | *>0.9999* | |
|  | Δ*fbiC* pRANGER-*fbiC* | | –1.41E+04 | –2.18E+05 | | to | | 1.90E+05 | | ns | | *>0.9999* | |
| Δ*fbiC* + 937 ng/mL F_O_ | Δ*fbiC* pRANGER-*fbiC* | | 1.85E+03 | –2.02E+05 | | to | | 2.06E+05 | | ns | | *>0.9999* | |

Abbreviations: SS: sum of squares, DF: degrees of freedom, MS: mean square, Mean Diff.: mean difference.

**References**

1. Komor AC, Kim YB, Packer MS, Zuris JA, Liu DR. Programmable editing of a target base in genomic DNA without double-stranded DNA cleavage. Nature. 2016;533(7603):420-4.

2. Jiang Y, Chen B, Duan C, Sun B, Yang J, Yang S. Multigene editing in the *Escherichia coli* genome via the CRISPR-Cas9 system. Appl Environ Microbiol. 2015;81(7):2506-14.

3. Li M, Huo YX, Guo S. CRISPR-mediated base editing: from precise point mutation to genome-wide engineering in nonmodel microbes. Biology. 2022;11(4):e571.

4. Blin K, Pedersen LE, Weber T, Lee SY. CRISPy-web: An online resource to design sgRNAs for CRISPR applications. Synth Syst Biotechnol. 2016;1(2):118-21.

5. Kuhn K, Baker SC, Chudin E, Lieu MH, Oeser S, Bennett H, *et al.* A novel, high-performance random array platform for quantitative gene expression profiling. Genome Res. 2004;14(11):2347-56.

6. Bolger AM, Lohse M, Usadel B. Trimmomatic: a flexible trimmer for Illumina sequence data. Bioinformatics. 2014;30(15):2114-20.

7. Lackner G, Moebius N, Partida-Martinez L, Hertweck C. Complete genome sequence of *Burkholderia rhizoxinica*, an endosymbiont of *Rhizopus microsporus*. J Bacteriol. 2011;193(3):783-4.

8. Solovyevand A, Salamov V. Automatic annotation of microbial genomes and metagenomic sequences. In: Li R, editor. Metagenomics and its applications in agriculture, biomedicine and environmental studies: Nova Science Pub Inc; 2011. p. 61-78.

9. Li C, Wen A, Shen B, Lu J, Huang Y, Chang Y. FastCloning: a highly simplified, purification-free, sequence- and ligation-independent PCR cloning method. BMC Biotechnol. 2011;11(1):92.

10. Shea A, Wolcott M, Daefler S, Rozak DA. Biolog phenotype microarrays. Methods Mol Biol. 2012;881:331-73.

11. Lackner G, Moebius N, Hertweck C. Endofungal bacterium controls its host by an hrp type III secretion system. ISME J. 2011;5(2):252-61.

12. Barrett AR, Kang Y, Inamasu KS, Son MS, Vukovich JM, Hoang TT. Genetic tools for allelic replacement in *Burkholderia* species. Appl Environ Microbiol. 2008;74(14):4498-508.

13. Doublet B, Douard G, Targant H, Meunier D, Madec JY, Cloeckaert A. Antibiotic marker modifications of lambda Red and FLP helper plasmids, pKD46 and pCP20, for inactivation of chromosomal genes using PCR products in multidrug-resistant strains. J Microbiol Methods. 2008;75(2):359-61.

14. Hossain MS, Le CQ, Joseph E, Nguyen TQ, Johnson-Winters K, Foss FW. Convenient synthesis of deazaflavin cofactor F_O_ and its activity in F_420_-dependent NADP reductase. Org Biomol Chem. 2015;13(18):5082-5.

15. Schindelin J, Arganda-Carreras I, Frise E, Kaynig V, Longair M, Pietzsch T, *et al.* Fiji: an open-source platform for biological-image analysis. Nat Methods. 2012;9(7):676-82.

16. Lackner G, Mobius N, Scherlach K, Partida-Martinez LP, Winkler R, Schmitt I, *et al.* Global distribution and evolution of a toxinogenic *Burkholderia*-*Rhizopus* symbiosis. Appl Environ Microbiol. 2009;75(9):2982-6.

17. Tomlin KL, Clark SR, Ceri H. Green and red fluorescent protein vectors for use in biofilm studies of the intrinsically resistant *Burkholderia cepacia* complex. J Microbiol Methods. 2004;57(1):95-106.

18. Niehs SP, Kumpfmüller J, Dose B, Little RF, Ishida K, Flórez LV, *et al.* Insect-associated bacteria assemble the antifungal butenolide gladiofungin by non-canonical polyketide chain termination. Angew Chem Int Ed. 2020;59(51):23122-6.
